# ESCRT-III/Vps4 Controls Heterochromatin-Nuclear Envelope Attachments

**DOI:** 10.1016/j.devcel.2020.01.028

**Published:** 2020-04-06

**Authors:** Gerard H. Pieper, Simon Sprenger, David Teis, Snezhana Oliferenko

**Affiliations:** 1 The Francis Crick Institute, 1 Midland Road, London NW1 1AT, UK; 2 Randall Centre for Cell and Molecular Biophysics, School of Basic and Medical Biosciences, King’s College London, London SE1 1UL, UK; 3 Institute for Cell Biology, Biocenter, Medical University of Innsbruck, Innrain 80/82, A-6020, Innsbruck, Austria

**Keywords:** nuclear envelope, ESCRT-III, Lem2, heterochromatin, fission yeast, Vps4

## Abstract

Eukaryotic genomes are organized within the nucleus through interactions with inner nuclear membrane (INM) proteins. How chromatin tethering to the INM is controlled in interphase and how this process contributes to subsequent mitotic nuclear envelope (NE) remodeling remains unclear. We have probed these fundamental questions using the fission yeast *Schizosaccharomyces japonicus*, which breaks and reforms the NE during mitosis. We show that attachments between heterochromatin and the transmembrane Lem2-Nur1 complex at the INM are remodeled in interphase by the ESCRT-III/Vps4 machinery. Failure of ESCRT-III/Vps4 to release Lem2-Nur1 from heterochromatin leads to persistent association of chromosomes with the INM throughout mitosis. At mitotic exit, such trapping of Lem2-Nur1 on heterochromatin prevents it from re-establishing nucleocytoplasmic compartmentalization. Our work identifies the Lem2-Nur1 complex as a substrate for the nuclear ESCRT machinery and explains how the dynamic tethering of chromosomes to the INM is linked to the establishment of nuclear compartmentalization.

## Highlights

• Nuclear compartmentalization after mitosis requires Lem2-Nur1 and ESCRT-III/Vps4
• ESCRT-III/Vps4 remodels links between Lem2 and heterochromatin in interphase
• Lem2 recruits ESCRT-III/Vps4 through Cmp7, but Vps4 pulls apart Lem2-Cmp7 complex
• Lem2-Nur1 release from chromatin enables nuclear membrane sealing on the spindle

## Teaser

Pieper et al. show that the inner nuclear membrane Lem2-Nur1 complex serves as a substrate for the nuclear ESCRT-III/Vps4 machinery. They find that dynamic tethering of chromosomes to the Lem2-Nur1 complex during interphase is linked to the establishment of post-mitotic nuclear compartmentalization in an emerging model fission yeast, *S. japonicus*.

## Introduction

Chromosomes are compartmentalized within the nucleus, which is delimited by the double membrane of the nuclear envelope (NE). Defects in the function and structure of the NE are linked to disease (Stewart et al., 2007, Ungricht and Kutay, 2017). During interphase, the NE organizes the genome and regulates its expression (Mekhail and Moazed, 2010). Interactions between the NE and chromatin are executed by inner nuclear membrane (INM) proteins such as members of the evolutionarily conserved chromatin-binding LEM (LAP2, Emerin, MAN1)-domain family (Anderson and Hetzer, 2007, Anderson et al., 2009, Barton et al., 2015, Harr et al., 2016). As cells enter mitosis, chromosomes are released from the NE and the NE is remodeled to form two daughter nuclei at mitotic exit (Güttinger et al., 2009). The fission yeast *Schizosaccharomyces japonicus* (*S. japonicus*) is an emerging model system to study mitotic NE breakdown and reassembly (Oliferenko, 2018) with highly stereotypic NE dynamics, including NE breakage and resealing at a single site (Aoki et al., 2011, Yam et al., 2011). Its genome encodes two LEM-domain proteins, Man1 and Lem2. In the related species, *S. pombe*, Lem2 and Man1 mediate chromatin-NE tethering during interphase (Gonzalez et al., 2012, Barrales et al., 2016), with Lem2 organizing heterochromatin through interactions with heterochromatin maintenance machinery (Barrales et al., 2016). We have previously shown that in *S. japonicus*, Lem2 and Man1 function at distinct stages of mitosis. Through interactions with anaphase chromosomes, Man1 coordinates partitioning of nuclear membrane and its constituents with chromosome segregation (Yam et al., 2013). The other LEM-domain protein, Lem2, has been implicated in promoting re-establishment of nucleocytoplasmic compartmentalization at the end of mitosis but the underlying mechanism remained unknown (Yam et al., 2011). In human cells, the function of the Lem2 ortholog in NE resealing was later proposed to be mediated by its recruitment of the membrane remodeling complex ESCRT-III (endosomal sorting complexes required for transport) together with the AAA-ATPase Vps4 (Olmos et al., 2015, Vietri et al., 2015, Gu et al., 2017). LEM-domain proteins were also proposed to work together with ESCRT-III/Vps4 in mediating nuclear pore complex (NPC) quality control in *Saccharomyces cerevisiae* (Webster et al., 2014, Webster et al., 2016) and maintaining NE integrity in *S. pombe* (Gu et al., 2017). These processes are thought to rely on the capacity of the ESCRT machinery to remodel membranes. If and how the function of Lem2 and ESCRT-III/Vps4 in establishing and maintaining nuclear compartmentalization is linked to their roles in chromatin organization remained unclear.

Combining cell biological and genetic analyses in *S. japonicus* with chromatin immunoprecipitation (ChIP) and *in vitro* reconstitution experiments, we now show that the ESCRT-III/Vps4 machinery remodels attachments between Lem2 and heterochromatin during interphase. This interphase function of ESCRT-III/Vps4 is required for the ensuing post-mitotic function of Lem2 and its interactor Nur1 in re-establishment of nuclear compartmentalization.

## Results

### Lem2-Nur1 Together with ESCRT-III/Vps4 Establish Nucleocytoplasmic Compartmentalization in the Presence of the Mitotic Spindle

**Figure 1:**
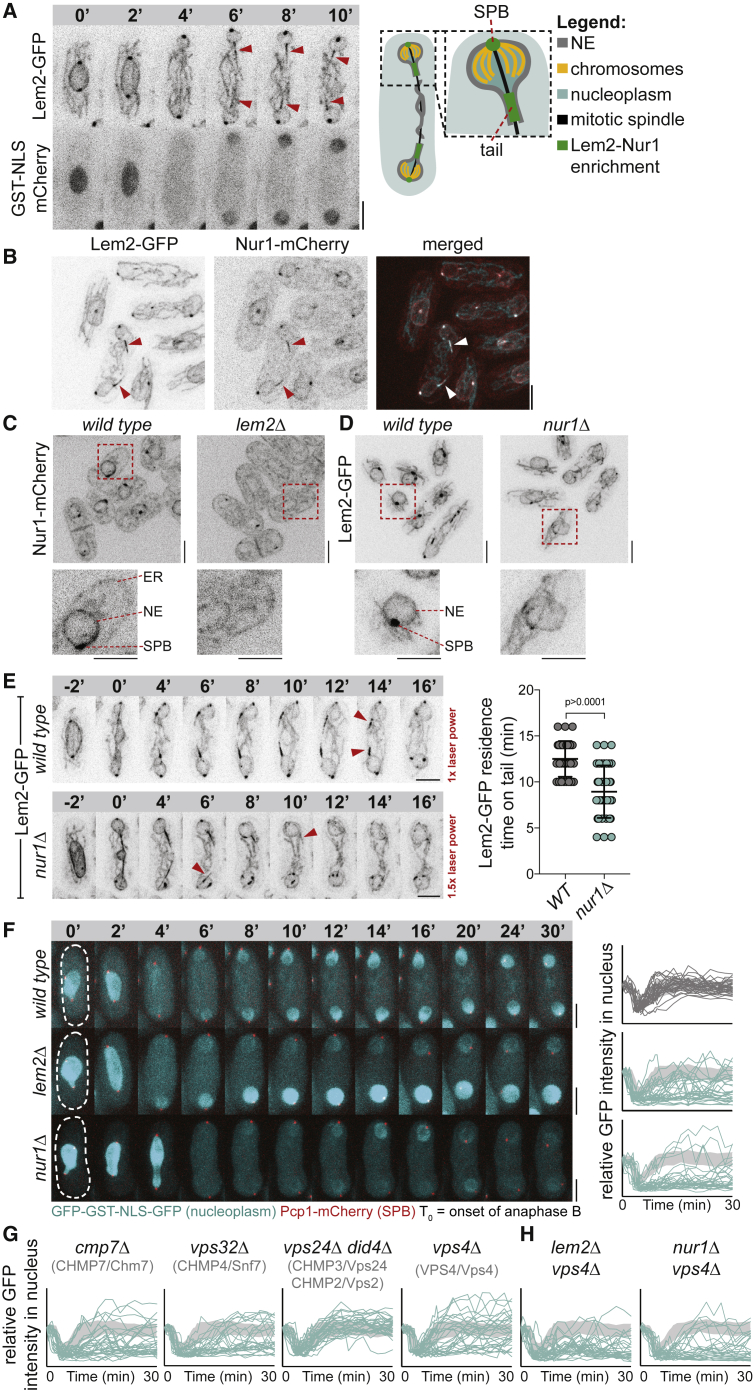
Lem2-Nur1 and a Minimal ESCRT-III/Vps4 Machinery Mediate NE Resealing in *S. Japonicus* (A) Time-lapse of a Lem2-GFP- GST-NLS-mCherry-expressing *S. japonicus* cell in anaphase. Arrowheads mark beginning of Lem2 enrichment at the “tails”. 8-min time point is shown schematically. (B) Cells co-expressing Lem2-GFP and Nur1-mCherry. Arrowheads indicate the “tails”. (C) Nur1-mCherry-expressing cells of indicated genotypes. See magnified images below. Shown are Z projections of 8 Z slices over a distance of 3.5 μm. (D) Z projections of images of Lem2-GFP-expressing cells. (E) Left, Lem2-GFP-expressing cells of indicated genotypes. The *nur1Δ* mutants were imaged using 1.5× higher laser power as compared to the WT. Arrowheads indicate the last time point before Lem2 disappearance from the “tail”. Right, quantification of the duration of Lem2 presence at the “tails”. Means and standard deviations (SD) are shown. p value determined by the Student’s t test. (F) Mitotic NE resealing assay in cells of indicated genotypes co-expressing GFP-NLS and Pcp1-mCherry. Quantification of the GFP fluorescence intensity in the nucleus is relative to the time point 0, prior to NE breakage (n = 15 cells/30 nuclei). SD for each time point in the WT (light gray) is shown together with the quantification of each mutant. (G) NE resealing assay for ESCRT-III/Vps4 mutants performed as in (F). (H) NE resealing assay performed for *lem2Δ vps4Δ* and *nur1Δ vps4Δ* double mutants as in (F). (A), (B), and (D–F) Shown are Z projections of spinning disc confocal stacks. (C and D) Cells imaged and presented at the same settings for comparison of signal strength. Scale bars represent 5 μm. See also Figure S1.

The establishment of nucleocytoplasmic compartmentalization in *S. japonicus* begins, while the spindle is still present (Yam et al., 2011). In wild-type (WT) cells, it occurs in a stereotypical manner 4–6 min after NE rupture in anaphase, signified by re-accumulation of the nucleoplasmic reporter protein GST-NLS-mCherry in the reforming daughter nuclei (Figure 1A). To allow compartmentalization at this stage, the nuclear membrane must be wrapped tightly around the intersecting spindle (Aoki et al., 2011, Yam et al., 2011). Lem2 enriches at these structures that we term “tails”, in addition to its spindle pole body (SPB) localization (Yam et al., 2011) (Figure 1A), suggesting that its localization to “tails” may support this process. Thus, nuclear compartmentalization is established prior to nuclear membrane resealing, which can only be completed after spindle breakdown.

Other NE proteins such as NPC components and the second fission yeast LEM-domain protein Man1 are largely excluded from the “tails” due to their interactions with segregating chromosomes at this stage of mitosis (Aoki et al., 2011, Yam et al., 2011, Yam et al., 2013). Thus, the “tail” represents a NE domain that is spatially segregated from the rest of the NE during mitotic exit. The regulated localization of INM proteins such as Lem2 to NE “tails” could be key for establishment of nuclear compartmentalization (Figure 1A, right panel).

Lem2 orthologs promote interphase heterochromatin tethering and heterochromatic gene silencing in complex with another INM protein, Nur1 (Mekhail et al., 2008, Banday et al., 2016, Barrales et al., 2016). In *S. japonicus*, Nur1 largely co-localized with Lem2, including enrichment at the NE “tails” (Figure 1B). This localization of Nur1 required Lem2, as Nur1 redistributed throughout the ER in *lem2Δ* cells (Figure 1C). Conversely, in cells lacking Nur1, less Lem2 signal was detected at the SPBs and the NE (Figure 1D). Additionally, Lem2 residence time at the “tails” was reduced in Nur1-deficient cells (Figure 1E). Thus, Lem2 and Nur1 depend on each other for proper localization to the NE and the SPB throughout the cell cycle.

We have previously shown that timely post-mitotic establishment of nucleocytoplasmic compartmentalization required Lem2 (Yam et al., 2011) (Figure 1F). Nur1 was equally required for this process. Whereas WT cells readily re-established nucleocytoplasmic compartmentalization, *lem2Δ* and *nur1Δ* mutants achieved this state considerably later and in a desynchronized manner (Figure 1F). These mutants also frequently failed to maintain nuclear integrity after a seemingly successful recovery event (Figure 1F). Following an extended delay, most mutant cells eventually recovered nuclear integrity. We concluded that the enrichment of the Lem2-Nur1 complex at the sites where the NE wraps around the mitotic spindle might be essential for the timely re-establishment of nucleocytoplasmic compartmentalization.

Given the links between LEM-domain proteins and ESCRT-III/Vps4 at the NE, we systematically addressed if ESCRT-III/Vps4 contributed to establishing nucleocytoplasmic compartmentalization in *S. japonicus*. Cells lacking the nuclear ESCRT-III adaptor Cmp7 (Vietri et al., 2015) (*S*. *c*: Chm7, *H*. *s*: CHMP7), the major ESCRT-III subunit Vps32 (Teis et al., 2008) (*S*. *c*: Snf7, *H*. *s*: CHMP4) and the AAA-ATPase Vps4 (Babst et al., 1997) exhibited defects in re-establishing nucleocytoplasmic compartmentalization following mitosis, similar to *lem2Δ* or *nur1Δ* cells (Figures 1G, S1A, and S1B). *lem2Δ vps4Δ* and *nur1Δ vps4Δ* double mutant cells exhibited phenotypes comparable to that of the single mutants (Figures 1H and S1C), suggesting that these proteins functioned in the same pathway. Two core ESCRT-III subunits, Vps24 (*S*. *c*: Vps24, *H*. *s*: CHMP3) and Did4 (*S*. *c*: Vps2, *H*. *s*: CHMP2), which are essential for endosomal ESCRT functions (Babst et al., 2002), were not required to establish nucleocytoplasmic compartmentalization (Figures 1G, S1A, S1B, and S1D). Similarly, the endosome-specific ESCRT-III adaptor, Vps25 (ESCRT-II), was not required for this process (Figure S1E).

Consistent with our earlier report of a premature loss of nucleocytoplasmic integrity in Lem2-deficient mitotic cells (Yam et al., 2011), we observed leaking of the nucleoplasmic marker in a subset of ESCRT-III/Vps4 mutants already at the onset of anaphase spindle elongation (Figure S1F), suggesting that the nuclei of these mutants might be prone to rupture. The persistence of the mitotic spindle was not affected by the lack of Lem2, Vps24, or Vps4 (Figure S1G), suggesting that ESCRT-III/Vps4-mediated establishment of nuclear compartmentalization and spindle breakdown are not coupled. We concluded that in *S. japonicus*, the Lem2-Nur1 complex works together with ESCRT-III subunits Cmp7-Vps32 and Vps4 to re-establish nuclear compartmentalization prior to the disassembly of the spindle.

**Figure 2:**
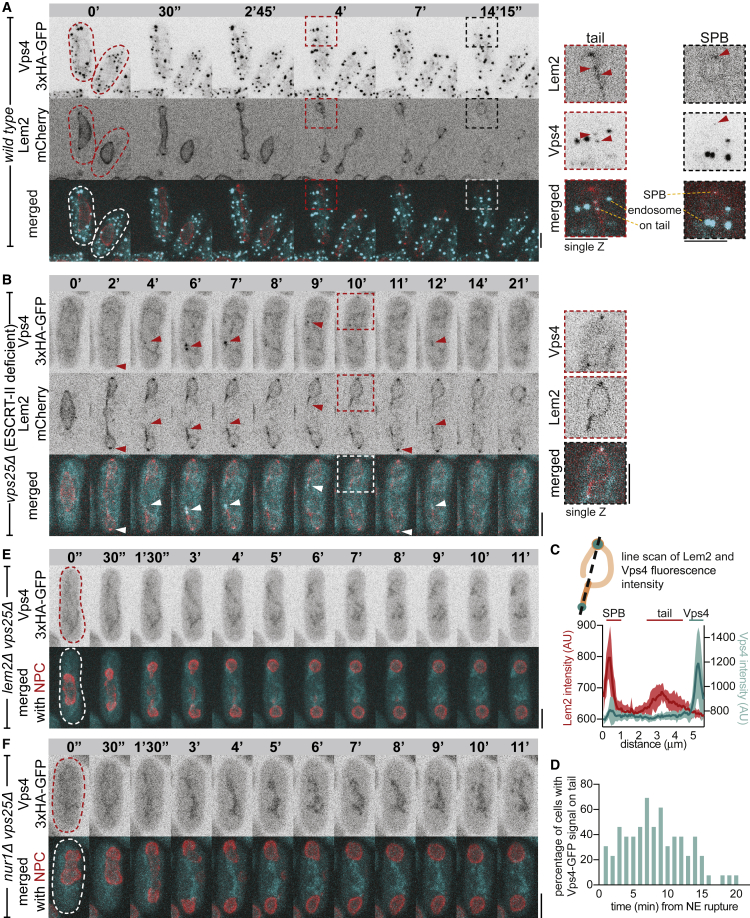
Lem2 Together with Nur1 Recruit ESCRT-III/Vps4 to NE Sealing Sites (A) Time-lapse maximum projection sequences of Vps4-GFP Lem2-mCherry-expressing cells starting prior to NE breakage. Magnified images (right) focus on a confocal slice showing Lem2 and Vps4 localization to the SPB and the “tail”. (B) A time-lapse Z-projected sequence of a *vps25Δ* Vps4-GFP Lem2-mCherry-expressing cell starting prior to NE rupture (n = 21 cells). Arrowheads indicate Vps4 localization to the SPBs and the distal end of the “tail”. A single Z slice of an area outlined at a 10-min time point shown as a magnified image on the right. (C) Quantification of Vps4-GFP signal at the distal end of the Lem2-mCherry “tail” (n = 10). (D) Quantification of the timing of Vps4 recruitment to the “tails”, shown as a percentage of cells exhibiting Vps4-GFP signal. Cells were followed between NE rupture and spindle breakdown (n = 13). (E) A time-lapse sequence of a *lem2Δ vps25Δ* Vps4-GFP Nup189-mCherry-expressing cell starting prior to NE rupture (n = 11). (F) A time-lapse sequence of a *nur1Δ vps25Δ* Vps4-GFP Nup189-mCherry-expressing cell starting prior to NE rupture (n = 17). (A), (B), (E), and (F) Z projections of spinning disc confocal stacks. Scale bars represent 5 μm. See also Figure S2.

The vast majority of functional, endogenously tagged Vps4-3xHA-GFP and Vps24-LAP-GFP (Figure S2A; referred to as Vps4-GFP and Vps24-GFP) was detected on cytoplasmic objects adjacent to FM4-64-marked vacuoles (Figures 2A and S2B), reminiscent of multivesicular bodies (MVBs) in budding yeast (Adell et al., 2017). Consistently, loss of endosomal adaptors *vps27* (ESCRT-0), *vps28* (ESCRT-I), and *vps25* (ESCRT-II) resulted in re-localization of Vps4-GFP from these perivacuolar objects into the cytoplasm (Figure S2B). Similarly, deletion of the ESCRT-III subunits *vps24* or *did4* resulted in mislocalization of Vps4-GFP to the cytosol (Figure S2B). In these mutants, only few Vps4-GFP objects were detected and they appeared to be distinct from perivacuolar MVBs.

With the majority of Vps4-GFP localizing to endosomes, it was difficult to detect Vps4 at the NE. Nevertheless, we observed Vps4-GFP localizing transiently to the Lem2 “tails” and the SPBs during mitotic exit (Figure 2A, arrowheads), the latter likely coinciding with SPB extrusion from the NE (Ding et al., 1997, Horio et al., 2002). We confirmed the localization of Vps4-GFP to the NE using a *vps25Δ* mutant where Vps4 no longer localized to endosomes (Figures 2B and S2B). In *vps25Δ* cells, Vps4-GFP was transiently recruited to the distal ends of the Lem2-mCherry-labeled NE “tails” (Figures 2B and 2C). Such highly dynamic behavior is evocative of dynamic ESCRT-III/Vps4 assemblies on endosomes (Adell et al., 2017, Wenzel et al., 2018), during HIV budding and cytokinesis in human cultured cells (Jouvenet et al., 2011, Mierzwa et al., 2017, Johnson et al., 2018). Vps4-GFP recruitment to tails was largely concurrent with re-establishment of nuclear compartmentalization (Figures 2D and 1A).

Vps4 recruitment to Lem2 “tails” depended on a functional Lem2-Nur1 complex. In the absence of Lem2 or Nur1, Vps4 was no longer detected at the NE (Figures 2E, 2F, and S2C). Vps4 recruitment also depended on Cmp7 and Vps32 (Figures S2D and S2E). Yet, the protein levels of full-length Vps4-GFP did not change in the absence of Vps25, Cmp7, or Lem2 (Figure S2F). Vps24-GFP exhibited similar localization to the NE “tails”, despite not being essential for re-establishment of nuclear integrity following NE breakdown (Figures S2G and S2H).

The timing of Vps4 recruitment to NE “tails” suggests that ESCRT-III/Vps4 functions to establish nucleocytoplasmic compartmentalization prior to spindle disassembly and subsequent membrane resealing. At this early stage ESCRT-III/Vps4 may play a role in the maintenance of the Lem2-Nur1 “tails”. In human cells, ESCRT-III is also recruited to the nuclear membrane in close vicinity of spindle remnants (Vietri et al., 2015), before they are dismantled allowing membrane closure.

### The AAA-ATPase Vps4 Disassembles Lem2-Cmp7 Interactions *In Vitro* and *In Vivo*

To analyze the interactions between Lem2 and the “minimal” ESCRT-III/Vps4 complex essential for nuclear re-compartmentalization, we set up *in vitro* assays using *S. japonicus* proteins purified from *Escherichia coli*. We purified the C-terminal nucleoplasmic domain of Lem2 (GST-Lem2^564-673^) encompassing the MAN1-Src1-C-terminal (MSC) domain, the Cmp7 C-terminal ESCRT-III-like domain (Cmp7^242-436^-3xFLAG), Vps32-3xMYC as well as Vps4-3xHA, and an ATP hydrolysis deficient Vps4^E233Q^3xHA substrate trap mutant that binds ATP but cannot efficiently hydrolyze it (Vps4^EQ^) (Babst et al., 1997). Purified Did4 was used as a positive control for Vps4 binding (Obita et al., 2007, Adell et al., 2014) (Figure S3A, lane 1). Vps4^EQ^ interacted directly with the ESCRT-III-like domain of Cmp7^242-436^ and Vps32 but not with Lem2^564-673^, suggesting that *in vitro* either ESCRT-III protein could recruit the substrate trap Vps4^EQ^ to Lem2 (Figure S3A).

**Figure 3:**
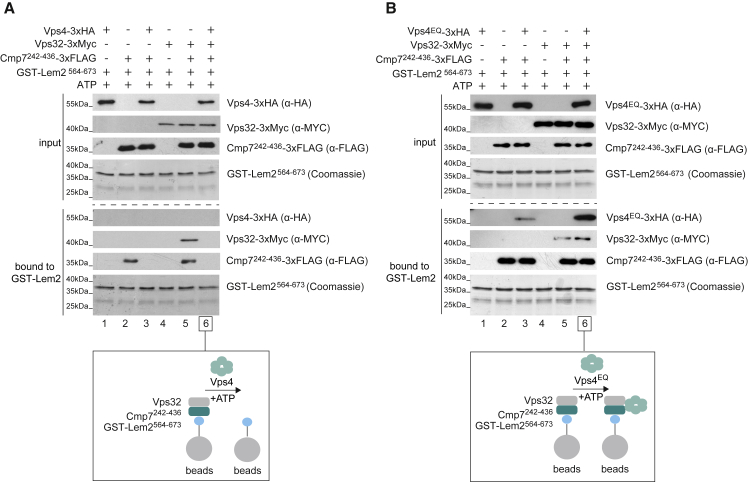
Vps4 Disassembles Lem2-Cmp7 Interactions *In Vitro* (A) *In vitro* binding assay. Upper panel, protein input with Coomassie staining for GST-Lem2^564-673^ and western blotting for ESCRT-III/Vps4. Lower panel, proteins bound to GST-Lem2^564-673^ on beads. A schematic overview of the experimental set-up and results is shown. (B) Same assay as in (A) but with the substrate trap Vps4^EQ^ mutant instead of the WT Vps4. See also Figure S3.

Lem2^564-673^ interacted directly with Cmp7^242-436^, indicating that the C-terminal MSC domain of Lem2 was sufficient for binding to the C-terminal ESCRT-III-like domain of Cmp7 (Figure 3A, lane 2). This was in agreement with previous findings in other organisms showing that the C terminus of Lem2 interacted with Cmp7 (Gu et al., 2017, Thaller et al., 2019). Direct binding of Vps32 to Lem2^564-673^ was not detected (Figure 3A; lane 4) and we observed only little binding between Vps32 and Cmp7^242-436^ in absence of Lem2 (Figure S3B). Yet, Vps32 was capable of associating with preformed Lem2^564-673^-Cmp7^242-436^ complexes (Figure 3A; lane 5). When Vps4 was added to the preformed Lem2^564^^-^^673^-Cmp7^242-436^-Vps32 complexes, it released Vps32 and Cmp7^242-436^ from Lem2^564-673^ (Figure 3A; lanes 3 and 6), in an ATP-dependent manner (Figure S3C; lane 1 and lane 3). The substrate trap mutant Vps4^EQ^ failed to disassemble Lem2^564-673^-Cmp7^242-436^ interaction and instead was retained on these complexes (Figure 3B; lane 3). Significantly more Vps4^EQ^ was trapped in complexes containing Vps32 in addition to Cmp7^242-436^ (Figure 3B; lane 6). We concluded that Vps4 could disassemble the interaction between Lem2 and Cmp7 *in vitro*, in an ATP-dependent manner.

**Figure 4:**
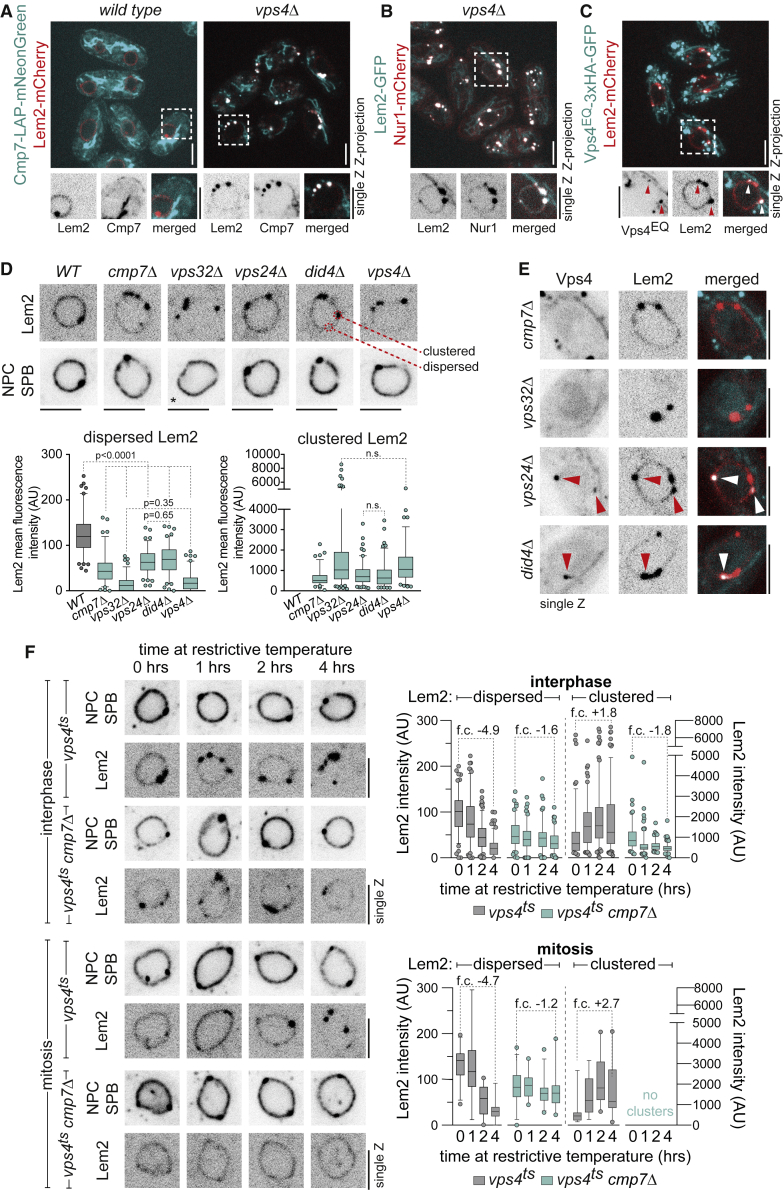
ESCRT-III/Vps4 Prevents Clustering of Lem2 at the NE (A) Cmp7-LAP-mNeonGreen and Lem2-mCherry in WT and *vps4Δ* cells. Cmp7 was faintly detected at the cortical ER in the WT; images show mitochondrial autofluorescence because of high exposure. (B) Lem2-GFP and Nur1-mCherry in *vps4Δ* cells. (C) Interphase cells expressing Vps4^EQ^-GFP and Lem2-mCherry. (D) (Top) Single confocal Z slices of nuclei of Lem2-mCherry Nup189-GFP Sad1-mNeonGreen-expressing cells of indicated genotypes. The *vps32Δ* cells expressed Lem2-mCherry and Nup189-GFP as we were not able to obtain Sad1-mNeonGreen in this genetic background (indicated by ^∗^). (Bottom) Quantifications of the dispersed and clustered Lem2-mCherry signal at the NE. N ≥ 95 nuclei per genotype. p values determined by Kruskal-Wallis test with Benjamini-Hochberg false discovery rate correction. (E) Single confocal Z slices of representative nuclei of Vps4-GFP Lem2-mCherry-expressing cells of indicated genotypes. Arrowheads indicate Lem2 NE clusters overlapping with Vps4 signal. (F) (Left) Time course showing the extent of Lem2-mCherry clustering in *vps4*^*ts*^-*gfp* and *vps4*^*ts*^*-gfp cmp7Δ* cells upon the shift to the restrictive temperature of 36°C (at 0, 1, 2, and 4 h). Nup189-GFP and Sad1-mNeonGreen mark the NE and the SPBs, respectively. (Right) Quantification of dispersed and clustered Lem2-mCherry signal, processed as in (D). Data for interphase and mitotic cells are shown (3 experiments, n ≥ 102 for interphase dispersed and clustered Lem2-mCherry signal, n ≥ 17 for mitotic dispersed signal, and n ≥ 13 for mitotic clustered signal). FC, fold change. (A–C) Maximum projections of spinning disc confocal stacks. Magnified confocal slices shown below. (A–F) Scale bars represent 5 μm. See also Figure S4.

Consistent with this model, in the absence of Vps4, Lem2-mCherry was no longer dispersed around the NE but instead was trapped in large clusters together with Cmp7-mNeonGreen (Figure 4A). In WT cells Cmp7-mNeonGreen was faintly visible around the cell cortex and sometimes at the NE (Figure 4A), indicating its ER localization as previously observed for the budding yeast and human Cmp7 proteins (Bauer et al., 2015, Olmos et al., 2016). The Lem2 NE clusters also contained Nur1 (Figure 4B). In line with our *in vitro* experiments, expression of Vps4^EQ^-GFP in WT cells caused the accumulation of Lem2 together with Vps4^EQ^ in aberrant clusters at the NE during interphase (Figure 4C). Yet, in contrast to *vps4*Δ, a fraction of Lem2 remained dispersed around the NE, suggesting that Vps4^EQ^ retained low residual ATPase activity. Vps4^EQ^-GFP was also enriched at endosomes and the distal ends of Lem2 “tails” and the SPBs during mitotic exit (Figure S4A).

Core ESCRT-III mutants but not earlier ESCRT-0, -I, and -II-deficient cells exhibited Lem2 clustering at the NE during interphase (Figures 4D and S4B). Analysis of the extent of Lem2 clustering revealed marked differences among the mutants. The strongest phenotype was observed in *vps32Δ* and *vps4Δ* cells, where almost all Lem2 was trapped in clusters (Figure 4D). The other ESCRT-III mutants (*cmp7Δ*, *vps24Δ*, and *did4Δ*) exhibited milder clustering, with more Lem2 distributed along the NE at steady state (Figure 4D).

Interestingly, in *vps24Δ* and *did4Δ* cells, Vps4 was enriched in small Lem2 clusters (Figure 4E). Yet, Vps4 was not detected in Lem2 clusters when Cmp7 or Vps32 were deleted (Figure 4E). We concluded that Lem2-Cmp7-Vps32 complexes were required for the recruitment of Vps4 to the NE. Vps24 and Did4 are not essential for Vps4 recruitment but are likely to stimulate Vps4 dynamics and function. Once recruited, Vps4 releases Cmp7 from Lem2, thus promoting dynamic interactions between the ESCRT-III machinery and Lem2. When this is not possible (either in *vps32Δ* or *vps4Δ* mutants), Lem2 and Cmp7 are persistently locked together in large clusters at the NE.

To analyze how Lem2 clusters arise, we acutely inactivated Vps4, using a temperature-sensitive mutant, Vps4^ts^-GFP (*vps4*^*I307T,L327S*^*-gfp*) (Babst et al., 1997). At 25°C, Lem2 localized to the SPBs and remained largely distributed around the NE, although we detected some clustering, indicating that Vps4^ts^-GFP was a mild hypomorph (Figure 4F). Upon shifting to the restrictive temperature of 36°C, Lem2 became increasingly trapped in NE clusters, which eventually persisted throughout mitosis (Figure 4F). We next used this inducible inactivation of Vps4 to explore how Cmp7 contributed to the formation of Lem2 clusters. During interphase, the lack of Cmp7 attenuated Lem2 cluster formation upon Vps4^ts^ inactivation, phenocopying Cmp7 loss of function. Remarkably, the formation of mitotically stable Lem2 clusters was strictly dependent on Cmp7, with all Lem2 clusters dispersing during mitosis in *cmp7Δ vps4*^*ts*^ cells at the restrictive temperature (Figure 4F). These results corroborate the hypothesis that Vps4 disassembles Lem2-Cmp7 complexes at the INM in interphase.

We tested if Lem2 clusters represented so-called storage of improperly assembled NPCs compartments (SINCs) described in budding yeast (Webster et al., 2014). However, we did not detect co-localization between Lem2 and two nucleoporins found in SINCs, Nup184 (*S. cerevisiae* Nup188) and Nup85 (*S. cerevisiae* Nup85) in *vps4Δ* mutants. Rather, nucleoporins were virtually excluded from Lem2 clusters (Figures S4C and S4D). Of note, we observed stable NE-associated foci of Vps4-GFP in a fraction of WT interphase cells (Figures S4E and S4F; typically, one Vps4-GFP object per nucleus). The formation of these Vps4-GFP assemblies was strictly dependent on Cmp7 and Lem2 (Figures S4E and S4F). Similar to Lem2 clusters, the Vps4-GFP NE foci were not enriched for nucleoporins (Figure S4E). Thus, the Lem2 clustering observed in ESCRT-III/Vps4-deficient *S. japonicus* is a distinct phenotype from the NPC assembly and storage defects suggested for budding yeast.

### ESCRT-III/Vps4 Releases Lem2 from Its Nur1-Mediated Attachments to Heterochromatin

**Figure 5:**
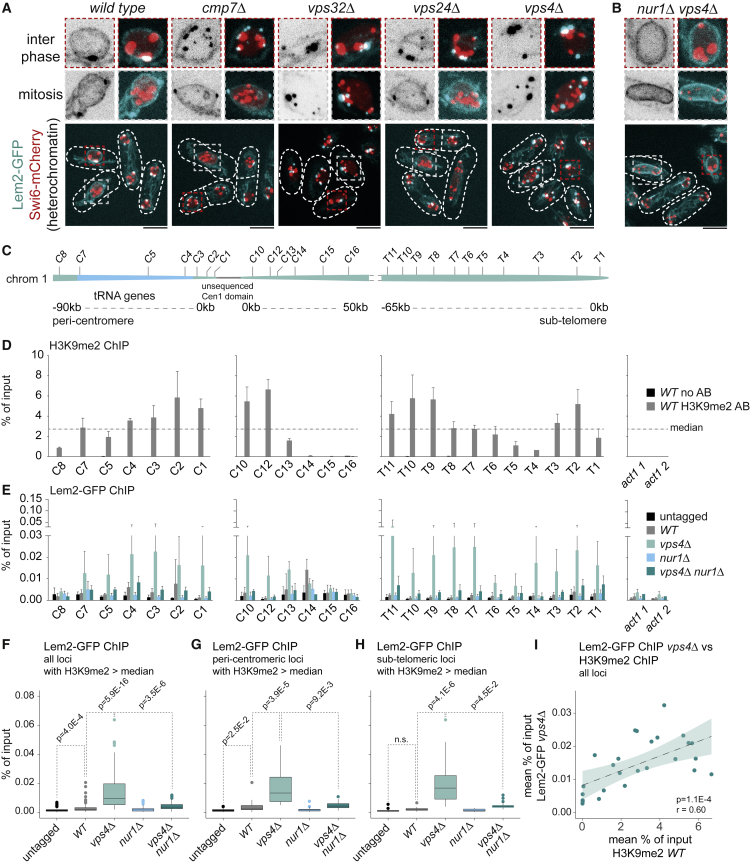
Vps4 Regulates Lem2-Heterochromatin Association (A) Maximum projections of spinning disc confocal stacks of cells of indicated genotypes co-expressing Lem2-GFP and Swi6-mCherry. Top, magnifications of interphase and mitotic cells. Scale bars represent 5 μm. (B) Same set-up as (A) in *nur1Δ vps4Δ* double mutants. (C) Scheme depicting the pericentromeric and subtelomeric regions of chromosome 1 of *S. japonicus* (not to scale). The locations of the qPCR primer sets are indicated. (D) ChIP-qPCR of H3K9me2 in WT cells (n = 3, means and standard deviations are shown). (E) ChIP-qPCR of Lem2-GFP in strains of indicated genotypes (n = 3) of the same genomic loci as in (D). (F) Box plots of the pooled Lem2-GFP ChIP-qPCR data of the genomic loci with higher than median H3K9me2 levels. p values determined by Kruskal-Wallis test with Dunn post-hoc test and Benjamini-Hochberg false discovery rate correction. (G and H) Same as in (E) but for the pericentromeric (G) and subtelomeric (H) regions. (I) Correlation plot of mean Lem2-GFP ChIP-qPCR signal versus the mean H3K9me2 ChIP-qPCR signal for all tested genomic loci. Dotted line corresponds to the linear trend line, shaded area corresponds to the 95% confidence interval. Pearson correlation coefficient r = 0.60 and p = 1.1E−4. See also Figure S5.

Since the Lem2-Nur1 complex is involved in heterochromatin maintenance and its tethering to the NE (Gonzalez et al., 2012, Banday et al., 2016, Barrales et al., 2016, Tange et al., 2016), we analyzed if the Nur1-Lem2 clusters in ESCRT-III-deficient cells co-localized with heterochromatin. In fission yeast, heterochromatin including subtelomeric and pericentromeric sequences localizes to the nuclear periphery during interphase (Allshire and Ekwall, 2015). Indeed, the interphase Lem2-GFP clusters that formed in ESCRT-III mutants were often adjacent to heterochromatic domains marked by either C-terminally or N-terminally tagged heterochromatin protein 1 (HP1) ortholog Swi6 (Figures 5A and S5A). Similarly, they often bordered the telomere marker Taz1 (Figure S5B). *S. japonicus*, like other eukaryotes, releases chromosomes from the INM as it enters mitosis (Hediger et al., 2002, Güttinger et al., 2009, Ebrahimi and Cooper, 2012, Fujita et al., 2012, Kanoh, 2013, Yam et al., 2013). Remarkably, the Lem2-heterochromatin clusters persisted throughout mitosis in *vps32Δ* and *vps4Δ* cells (Figures 5A and S5B). This suggested that Lem2-heterochromatin attachments in these mutants were refractory to mitotic regulation responsible for chromatin release (Güttinger et al., 2009), likely because Cmp7 and Lem2 were trapped together. Consistent with this hypothesis, Cmp7 was essential for the formation of mitotically stable clusters as Lem2 dispersed throughout the NE not only in mitotic *cmp7Δ* but also in *vps4*^*ts*^ *cmp7Δ* mutants (Figures 4F, 5A, and S5B). Thus, Cmp7 plays an integral role in the formation of persistent heterochromatin-associated Lem2-Nur1 clusters in the absence of Vps4 activity. De-clustering of Lem2 during mitosis in *vps24Δ* cells (Figures 5A and S5B) is likely due to the fact that Vps4 is still recruited to the NE in the absence of Vps24 and is capable of residual function (Figure 4E).

The LEM/HEH (helix extension helix) domain of *S. pombe* Lem2 was proposed to bind to DNA and mediate centromere tethering to the SPB, whereas the C-terminal MSC domain anchors telomeres and is required for heterochromatin silencing (Barrales et al., 2016, Hirano et al., 2018). The *S. japonicus* mutant of Lem2 lacking the N-terminal LEM/HEH domain (Lem2^ΔHEH^) exhibited normal localization to the nuclear periphery and SPBs in the WT and clustered with heterochromatin in cells lacking ESCRT-III/Vps4 activity (Figure S5C). This suggests that heterochromatin interactions are not mediated through the LEM/HEH domain of Lem2 and that the ESCRT-III/Vps4 system does not act through this region of the protein. However, association of Lem2 with heterochromatin in *vps4Δ* mutant cells was prevented in the absence of Nur1, indicating that Nur1 linked Lem2 to heterochromatin (Figures 5B and S5D).

Our imaging experiments suggested that ESCRT-III/Vps4-dependent disassembly of Lem2-Cmp7 interactions was linked to the release of Lem2-Nur1 complexes from heterochromatin. To test this hypothesis, we have performed ChIP-qPCR on Lem2-GFP. *S. japonicus* centromeres have a markedly different structure than those of *S. pombe*. They do not have stereotypic central core domains but rather, consist of heterochromatinized retrotransposons (Tong et al., 2019). The current genome assembly does not include the centromeres themselves but the pericentromeres have been assembled (Rhind et al., 2011), allowing us to design an extensive panel of primer pairs covering the pericentromeric regions and the right subtelomere of chromosome I (Figure 5C).

We mapped heterochromatin at these regions in WT cells by determining the extent of H3K9me2 enrichment (Allshire and Ekwall, 2015). Consistent with the pattern of heterochromatinization in *S. pombe* (Kanoh et al., 2005), we detected varying levels of H3K9me2 enrichment throughout the subtelomere. We also detected H3K9me2 around the centromere of chromosome 1 (Figures 5C and 5D). H3K9me2 covered the 75 kb tRNA-rich region on the left chromosome arm and spread approximately 15 kb away from the central centromere domain on the right chromosome arm (Figure 5D). As expected, we did not detect heterochromatin on the gene encoding actin, which is found in a gene-dense region of chromosome 2 (Figure 5D).

We detected a stronger enrichment of Lem2-GFP at highly heterochromatinized regions (e.g., C10 or T11) in *vps4Δ* cells, as compared to the WT, *nur1Δ* or *vps4Δ nur1Δ* mutants (Figure 5E), but not in euchromatic regions (e.g., the actin gene) (Figure 5E). Immunoprecipitated Lem2-GFP protein levels were similar in all tested genotypes (Figure S5E). To infer the overall effect of the specific genotype on the strength of Lem2-heterochromatin interactions, we pooled the Lem2-GFP ChIP-qPCR results at loci with high levels of heterochromatinization, defined as those with higher than median H3K9me2 levels (Figures 5E and 5F). We also performed the same analysis specifically for the pericentromeric (Figure 5G) and subtelomeric regions (Figure 5H). In the WT, we detected very mild enrichment of Lem2 at the heterochromatin around the centromere (Figures 5F–5H). However, the recovery of Lem2 at both pericentromeric and subtelomeric heterochromatin was markedly increased in the *vps4Δ* mutant (Figures 5F–5H). The enrichment of Lem2 at heterochromatin was correlated to H3K9me2 levels (Figure 5I, Pearson correlation coefficient r = 0.6). This Lem2 enrichment depended on Nur1, consistent with its function in linking Lem2 to heterochromatin (Figures 5F–5H).

Taken together, our experiments show that ESCRT-III/Vps4 maintains transient interactions of Lem2-Nur1 with heterochromatin and that the loss of Vps4 activity locks Lem2-Nur1 complexes at heterochromatin.

**Figure 6:**
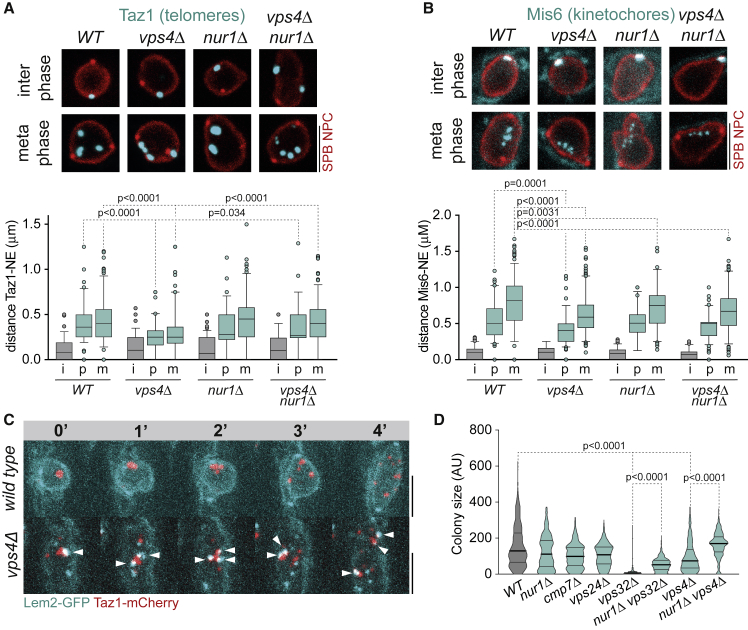
ESCRT-III/Vps4-Mediated Remodeling of Lem2-Heterochromatin Association Is Required for the Release of Chromosome Arms from the NE during Mitosis (A) (Top) Representative Z projections of the middle 8 confocal slices of nuclei of Taz1-GFP Nup189-mCherry Pcp1-mCherry expressing cells of indicated genotypes. I, interphase; P, prometaphase; M, metaphase. (Bottom) Quantifications of telomere-NE distance. p values determined by Kruskal-Wallis test and Benjamini-Hochberg false discovery rate correction, performed independently for prophase and metaphase cells. (B) Same set-up as (A) for the kinetochore (labeled by MIS6-GFP)-NE distance. (C) Time-lapse maximum projection sequences of WT and *vps4Δ* cells expressing Lem2-GFP and Taz1-mCherry, focusing on mitotic nuclei. Arrowheads indicate the fate of a Lem2-associated telomere cluster. (D) Quantification of the colony size of the pooled data of 3 technical replicates of 3 biological experiments. p values determined by Kruskal-Wallis test and Benjamini-Hochberg false discovery rate correction. (A–C) Scale bars represent 5 μm. See also Figure S6.

We characterized the fate of the major heterochromatin domains during mitosis in *vps4Δ* cells where the Lem2-Nur1 complex forms stable attachments to heterochromatin. As expected from the release of chromosome arms from the NE during early mitosis in the WT (Fujita et al., 2012, Yam et al., 2013), the distance between Taz1-GFP and Nup189-mCherry increased already in prometaphase (Figure 6A). However, most telomeres remained adjacent to the NE in mitotic *vps4Δ* cells (Figure 6A). When we disrupted the link between Lem2 and heterochromatin by additionally removing Nur1, the telomere release from the NE was restored (Figure 6A). Kinetochores of *vps4Δ* cells labeled by Mis6-GFP detached from the NE, although the kinetochore-NE distance was reduced as compared to the WT (Figure 6B). Their movement might be spatially constrained, likely as a consequence of failed telomere release. Kinetochore-NE distance was also decreased in the absence of Nur1, possibly due to nuclear shape abnormalities (Figure 6B).

Consistent with the failed release of subtelomeric heterochromatin from Lem2, telomeres remained associated with Lem2 clusters throughout mitosis in *vps4Δ* mutants (Figures 6C and S6A). The persistent telomere-NE association deregulated mitotic progression in *vps4Δ* cells. In the WT, the spindle assembly checkpoint (SAC) protein Mad2-GFP (Musacchio, 2015) typically remained on kinetochores for 5–10 min (Figure S6B). However, in *vps4Δ* cells, we observed an abnormally broad distribution of the duration of SAC activation. Whereas some cells were delayed at the metaphase-to-anaphase transition, others had a marked decrease in duration of Mad2 kinetochore recruitment (Figure S6B).

We observed severe growth defects only in the ESCRT-III/Vps4 mutants that exhibited persistent heterochromatin-NE associations (*vps32Δ* and *vps4Δ*). They formed fewer (Figure S6C, bottom panel) and smaller colonies (Figures 6D and S6C, top panel). The size of the colonies was rescued to a large extent by breaking up persistent Lem2-heterochromatin interactions by additionally deleting *nur1* (Figure 6D). This occurred despite defective nucleocytoplasmic compartmentalization in the *nur1Δ vps4Δ* double mutant (Figure 1H). Our results may explain the growth defect associated with *vps4* deletion in *S. pombe*, which was rescued by additionally deleting *lem2* or *cmp7* (Gu et al., 2017). Indeed, in the absence of Vps4 in *S. pombe*, Lem2 exhibited severe clustering throughout mitosis (Figure S6D), suggesting that ESCRT-III/Vps4 function in preventing Lem2-heterochromatin clustering is evolutionarily conserved. We concluded that persistent heterochromatin association with Lem2-Nur1 at the NE was a major cause of substantial growth defects in Vps32- and Vps4-deficient cells.

### ESCRT-III/Vps4-Mediated Release of Lem2 from Heterochromatin Is Required for Its Function in Nucleocytoplasmic Compartmentalization at Mitotic Exit

**Figure 7:**
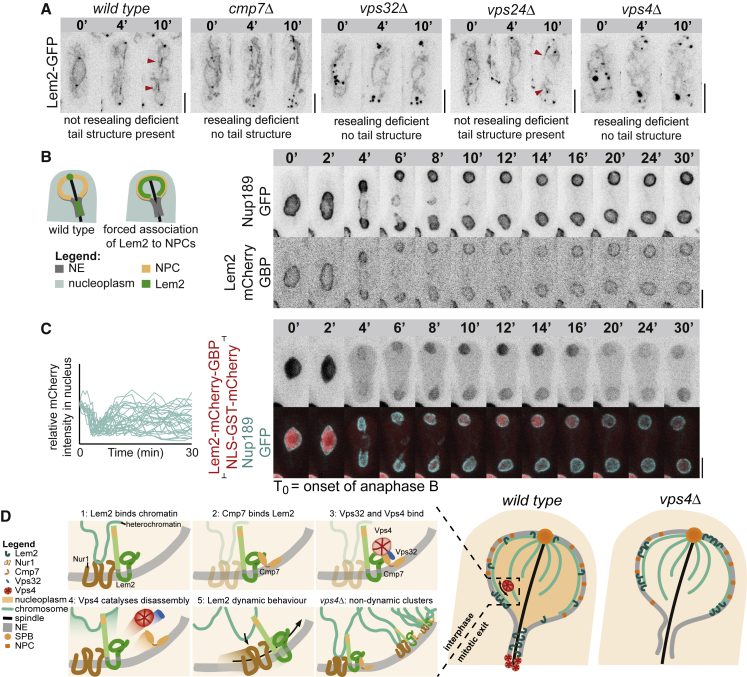
Lem2 Must Relocalize to the NE “Tails” to Ensure Nucleocytoplasmic Compartmentalization at Mitotic Exit (A) Maximum projections of time-lapse spinning disc confocal stacks of cells of indicated genotypes expressing Lem2-GFP starting from prior to NE breakdown. Arrowheads indicate the presence of “tail” structures. (B) Left, scheme of the synthetic tethering of Lem2 to Nup189 that prevents its enrichment at the NE “tails”. Right, time-lapse sequence of a cell co-expressing Lem2-mCherry-GBP and Nup189-GFP. (C) Left, quantifications of relative mCherry intensity in the nuclei of 15 cells (30 nuclei). Right, a representative cell exhibiting a NE resealing phenotype. (D) A cartoon of our model for ESCRT-III/Vps4 function in interphase chromatin tethering to the NE and the resulting NE sealing phenotypes during mitosis. (A–C) Scale bars represent 5 μm.

We hypothesized that release of Lem2 from heterochromatin was required for Lem2 to enrich at the NE “tails” at the end of mitosis. Indeed, in the mutants that were deficient in nuclear re-compartmentalization (*nur1Δ*, *cmp7Δ*, *vps32Δ*, and *vps4Δ*) Lem2 failed to enrich on “tail” structures (Figure 7A; compare with Figure 1E). It likely occurs for different reasons: in *nur1Δ* and *cmp7Δ* mutants, Lem2 is not retained at the “tails,” whereas in *vps32Δ* and *vps4Δ* mutants Lem2 did not detach from heterochromatin and therefore could not relocalize to the “tails”. Of note, in the *vps24Δ* mutant that was not defective in nuclear compartmentalization, Lem2 was still present at the “tails” (Figure 7A). To test the hypothesis that Lem2 sequestration at heterochromatin prevents its function in nuclear compartmentalization, we generated a Lem2 version that could not reach the NE “tails”. To this end, we tagged Lem2 with GFP-binding protein (GBP) and expressed it together with Nup189-GFP, forcing the binding of Lem2 to the NPCs (Figure 7B). The NPCs are excluded from the “tail” domain and instead are associated with chromosome arms through Man1 (Yam et al., 2013) (Figure 7B). Indeed, Lem2-GBP/Nup189-GFP complexes localized around the NE but were absent from “tails” (Figure 7B). Failure to localize Lem2 to the regions where the spindle intersects with the NE led to a marked deficiency in re-establishing nucleocytoplasmic compartmentalization (Figure 7C). Under these conditions, ESCRT-III and Vps4 were fully functional, but Lem2 was sequestered away from the “tails”. Hence the function of ESCRT-III/Vps4 machinery in this process depended critically on the localization of Lem2-Nur1 to NE “tails”.

We conclude that interphase ESCRT-III/Vps4-dependent remodeling of Lem2-Nur1 complexes with heterochromatin allows its timely enrichment at NE “tails” to establish a seal at the site where the spindle intersects with the nuclear membrane. This process promotes re-establishment of nucleocytoplasmic compartmentalization at the end of mitosis.

## Discussion

Our findings define a mechanism through which the ESCRT-III/Vps4 machinery controls tethering of heterochromatin to the Lem2-Nur1 complexes during interphase. We propose the following model (Figure 7D): (1) the Lem2-Nur1 complex binds to heterochromatin; (2) this enables Cmp7 interaction with Lem2; (3) Cmp7 then recruits Vps32 and Vps4, which (4) work together to catalyze the release of Cmp7 from Lem2. We speculate that this remodeling step (5) concurrently promotes the detachment of Lem2 from heterochromatin.

Both *in vitro* and genetics data suggest that Cmp7 serves as an adaptor bringing the rest of the ESCRT-III/Vps4 machinery to remodel Lem2-heterochromatin interactions at the INM. In the absence of Cmp7, the entire complex is absent, stabilizing Lem2 association with heterochromatin, which manifests as a Lem2 clustering phenotype that can be resolved during mitosis.

In the absence of the ESCRT-III subunit Vps32, Vps4 cannot be recruited to the NE to release the interactions between Lem2 and Cmp7. Therefore, Lem2 remains locked with Cmp7 on heterochromatin. Finally, in the absence of Vps4, the interaction between Lem2 and Cmp7-Vps32 is also not disassembled, phenocopying *vps32* deletion. Thus, it appears that once bound to Lem2, Cmp7 must be removed by Vps4 from this complex. If this is not possible, the presence of Cmp7 exacerbates the heterochromatin-locked state of Lem2-Nur1. The detrimental role of accumulating Cmp7 at the NE was also suggested for other organisms (Thaller et al., 2019, Vietri et al., 2019, Gu et al., 2017).

Vps24 and Did4 appear to support but are not strictly necessary for Vps4 recruitment and function at the INM. In *vps24*Δ and *did4*Δ mutants, Lem2 clusters mildly at heterochromatin but Vps4, which is enriched in these clusters, appears to resolve them during mitosis. The non-essential role of Vps24 and Did4 in supporting Vps4 function at the NE is underscored by the lack of strong NE resealing phenotypes in the respective *S. japonicus* mutants. These results imply that ESCRT-III assemblies consisting of Cmp7, Vps32, and Vps4 are sufficient to support post-mitotic nuclear compartmentalization but might not be able to properly execute other functions of the nuclear ESCRT machinery.

Vps4 remodels Lem2-Nur1 interactions both with pericentromeric and subtelomeric heterochromatin. However, the mitotic outcomes of Vps4 deficiency for these chromosome regions are markedly different. Only telomeres are retained at the NE when Vps4 fails to disassemble Lem2-Cmp7 complexes. Such a distinct behavior may be explained by centromeres being connected to the SPBs rather than directly with the INM (Fernández-Álvarez et al., 2016). ESCRT-III dysfunction has been associated with nuclear membrane deformations, possibly through unchecked assembly of Cmp7 (Webster et al., 2014, Thaller et al., 2019, Vietri et al., 2019, Gu et al., 2017). We speculate that Cmp7-dependent persistent heterochromatin-INM association may lead to INM deformations where the Lem2-heterochromatin complexes are sequestered and become refractory to mitotic signaling. Whereas this would affect the INM-associated subtelomeric heterochromatin, the SPB-tethered centromeres would be protected. Additionally, the SPBs function as a platform for mitotic signaling (Fernández-Álvarez et al., 2016), suggesting that the physical proximity to them may impact on the efficiency of chromatin release from the NE.

The regulation of Lem2-heterochromatin attachments during interphase by ESCRT-III/Vps4 is prerequisite for the bulk release of chromosomes from the NE at mitotic entry. This frees up Lem2 and Nur1 to execute their post-mitotic function in organizing the NE sealing sites around the mitotic spindle (Figure 7D). Once at the NE “tails”, Lem2-Nur1 complexes again engage ESCRT-III/Vps4 to promote re-establishment of nucleocytoplasmic compartmentalization. This initial re-compartmentalization occurs in the presence of the spindle, indicating that this ESCRT-III/Vps4-dependent step does not involve resealing of the NE membrane. Both the timing of Vps4 recruitment to the “tails” and the fact that the nuclei fail to re-compartmentalize in the presence of the spindle in Vps4-deficient cells are consistent with this earlier function. Of note, despite a delay, the daughter nuclei eventually reseal in ESCRT-III- and Vps4-deficient cells, both in *S. japonicus* (Figure 1) and human tissue culture (Olmos et al., 2015, Vietri et al., 2015). Similarly, re-establishment of nuclear compartmentalization upon nuclear ruptures induced by migration through confined spaces is delayed but not abrogated in cells deficient in ESCRT-III function (Denais et al., 2016, Raab et al., 2016). This may indicate that nuclear membrane fusion could rely on pathways parallel to ESCRT-III/Vps4. Alternatively, the ESCRT-III/Vps4 machinery at the NE may indeed perform functions that are distinct from nuclear membrane fusion. For instance, the repair of interphase laser-induced NE ruptures can occur independently of the ESCRT-III NE adaptor Chmp7 but requires recruitment of LEM-domain proteins to chromatin through BAF (Halfmann et al., 2019).

During mitotic exit Cmp7 might function through stabilizing the Lem2-Nur1-organized NE “tail” and together with ESCRT-III and Vps4 seal the membrane onto the spindle. Indeed, human Lem2-Cmp7 assemblies were proposed to act as a molecular sealant aiding nuclear membrane attachment to spindle microtubules (von Appen et al., 2019). Of note, the Lem2-Nur1 complex requires Cmp7 to enrich at the NE “tails” around the spindle to establish nucleocytoplasmic compartmentalization (Figure 7A).

The release of chromosomes from the NE, whether through breakdown of the NE or dissociation from an intact membrane, is a common feature of mitosis in eukaryotes (Hediger et al., 2002, Ebrahimi and Cooper, 2012, Fujita et al., 2012, Kanoh, 2013, Yam et al., 2013). Indeed, in human cells, the artificial tethering of histones to ER membranes leads to failure in chromosome segregation (Champion et al., 2019). Similarly, NE tethering of a single chromosomal locus leads to mis-segregation of this chromosome in budding yeast (Chen, 2019). Our data indicate that ESCRT-III/Vps4 remodels heterochromatin at the INM during interphase and this process is critical for proper progression through mitosis. It will be interesting to see if Vps4-assisted INM-chromatin remodeling contributes to (1) remodeling of Lem2D/BAF complexes during interphase and (2) post-mitotic reformation of the NE in metazoan cells, where membrane spreads around the genome through a Brownian ratchet-like mechanism. Importantly, the dynamicity of chromatin-NE interactions afforded by ESCRT-III/Vps4 may provide a yet to be explored regulation modality shaping interphase genome organization and function.

## STAR★Methods

### Key Resources Table

**Table:** 

REAGENT or RESOURCE	SOURCE	IDENTIFIER
**Antibodies**		

Anti-FLAG	Sigma	Cat. # 3165
Anti-MYC	Sigma	Cat. # 4439
Anti-3HA	Cell Signaling	Cat. # C29F4
Anti-GFP	Roche	Cat. # 11814460001
IRDye 800CW Goat anti-Mouse IgG	LI-COR	Cat. # 926-32210
GFP-Trap Magnetic agarose	Chromotek	Cat. # gtma-20
Anti-GFP antibody	Sigma-Aldrich	Cat. # 1814460001
Anti-H3K9me2 antibody	Abcam	Cat. # ab1220-100ug

**Bacterial and Virus Strains**		

C41(DE3)pLysS E.Coli	Lucigen	Cat. # 60444-1-LU
Subcloning Efficiency™ DH5™ Competent Cells	Invitrogen GmbH	Cat. # 18265-017

**Chemicals, Peptides, and Recombinant Proteins**		

ATP	Sigma-Aldrich	Cat. # A-2383
IPTG	Thermo Scientific	Cat. # R0392
Glutathione Sepharose 4B	GE Healthcare	Cat. # GE17-0756-01
L-Glutathione reduced min99%	Sigma	Cat. # G4251-25G
PreScission protease	GE Healthcare	Cat. # 27084301
Slide-a-lyzer	VWR	Cat. # 514-0172
Pierce magnetic glutathione beads	Thermo	Cat. # 78602
BSA Fraction V	Roth	Cat. # 8076.5
GST-Lem2 cterm	This Study	N/A
GST-Did4	This Study	N/A
GST-Vps32	This Study	N/A
GST-Cmp7	This Study	N/A
GST	This Study	N/A
Vps32-3xMYC	This study	N/A
Cmp7-3xFLAG	This Study	N/A
Vps4-3xHA	This Study	N/A
Vps4-EQ-3xHA	This study	N/A
Odyssey blocking buffer (TBS)	LI-COR	Cat. # 927-50000
NuPage MOPS SDS running buffer	Invitrogen	Cat. # NP0001
NuPage transfer buffer	Invitrogen	Cat. # NP0006-1
Revert 700 Total Protein Stain Kit	LI-COR	Cat. # 926-11010
NuPage LDS sample buffer	Invitrogen	Cat. # NP0007
Formaldehyde	Fisher Scientific	Cat. # BP531-25
AEBSF	Melford	Cat. # A20010-50
Leupeptin	Generon	Cat. # 103476-89-7
Protease inhibitor mix	Roche	Cat. # 25178600
Zirconium beads	MP biomedicals	Cat. # 6960-500
Protein-G dynabeads	Invitrogen	Cat. # 10003D
Proteinase K	New England Biolabs	Cat. # P8107S
ChIP DNA cleanup kit	Zymo Research	Cat. # #D5201
SyGreen Blue Mix Hi-ROX	PCR Biosystems	Cat. # PB20.16-20
Powerup SYBR Green mastermix	Applied Biosystems	Cat. # A25742
MicroAmp Plate Optical 384-Well	Applied Biosystems	Cat. # 4483315
MicroAmp Optical Adhesive Film	Applied Biosystems	Cat. # 4360954
Canavanine	Sigma	Cat. # C1625-250MG
FM4-64	Thermo Fisher	Cat. # T3166

**Experimental Models: Organisms/Strains**		

All *S. japonicus* and *S. pombe* strains are listed in Table S1	This study	N/A

**Oligonucleotides**		

All oligonucleotides are listed in Table S2	This study	N/A

**Recombinant DNA**		

Vps24-LAP-eGFP	Eurofins	Custom synthesis
Vps4-3xHA-eGFP	Eurofins	Custom synthesis
Cmp7-LAP-eGFP	Eurofins	Custom synthesis
pGex 6P1 GST-Lem2^564-673^	This study	N/A
pGex 6P1 GST-Did4	This study	N/A
pGex 6P1 GST-Cmp7^242-436^	This study	N/A
pGex 6P1 GST-Cmp7^242-436^-3xFLAG	This study	N/A
pGex 6P1 Vps4-3xHA	This study	N/A
pGex 6P1 Vps4^E233Q^-3xHA	This study	N/A
pGex 6P1 Vps32-3xMyc	This study	N/A

**Software and Algorithms**		

Adobe Photoshop CS6	Adobe Studios	N/A
Adobe Illustrator CS6	Adobe Studios	N/A
ImageJ/Fiji	Schindelin et al., 2012, Schindelin et al., 2017	https://imagej.net/Fiji
Quantstudio 6-7 Flex Studio	Applied Biosystems	N/A
R	(R Core Team, 2019)	https://www.R-project.org/
Excel	Microsoft	N/A
Primer3	Rosen and Skaletsky, 1998	http://biotools.umassmed.edu/bioapps/primer3_www.cgi
Prism 7/Prism 8	GraphPad Software	N/A

### Lead Contact and Materials Availability

Further information and requests for resources and reagents should be directed to and will be fulfilled by the Lead Contact, Snezhana Oliferenko (snezhka.oliferenko@crick.ac.uk). All unique/stable reagents generated in this study are available from the Lead Contact without restriction.

### Experimental Model and Subject Details

*S. japonicus* and *S. pombe* strains used in this study are listed in Table S1. Standard fission yeast methods and media were used (Moreno et al., 1991, Furuya and Niki, 2009, Aoki et al., 2010, Petersen and Russell, 2016). *S. japonicus* and *S. pombe* cells were typically maintained on yeast extract with supplements (YES) rich medium 2% agar plates at 24°C, 25°C or 30°C depending on the strains or experiment. For experiments, cells were grown to early exponential growth phase (OD_595 ∼_ 0.2–0.4) in liquid YES in baffled flasks in a shaker incubator at either 24°C, 25°C, 30°C or 36°C at 200 rpm. Typically, cells were pre-cultured overnight and then sub-cultured again the following morning. *S. japonicus* and *S. pombe* cells were mated on SPA agar plates and dissected on YES agar plates using a dissection microscope (MSM 400, Singer Instruments).

### Method Details

#### Molecular Genetics

All primers are shown in Table S2. Gene deletions and taggings were obtained using a plasmid-based or a PCR-based method and homologous recombination. All tagged proteins were tagged at their endogenous loci, with expression driven by the endogenous promoters, except when noted. For tagged constructs, partial open reading frames (ORFs) and regions downstream of genes of interest were cloned into pJK210-based plasmids containing either mCherry (pSO730 for *S. japonicus*) or eGFP (pSO729 for *S. japonicus*; pSO32 for *S. pombe*) and the full-length *S. japonicus* or *S. pombe ura4*^*+*^ gene. The *S. japonicus* Lem2-GFP construct was cloned into a pJK210-based plasmid carrying the *kanMX* resistance cassette (pSO820). For plasmid-based gene deletions, targeting constructs were made by cloning regions flanking the gene of interest into pJK210-based plasmids containing *kanR* (pSO820 for *S. japonicus*) or *natR* (pSO893 for *S. japonicus*) resistance cassettes, or the respective *ura4*^*+*^ genes (pSO550 for *S. japonicus*; pSO13 for *S. pombe*). PCR-based knockouts were obtained by amplifying the *hygR* resistance cassette flanked by 80 base pairs flanking the target gene. Plasmids were linearized before transformation. Transformation of *S. japonicus* was done by electroporation (Aoki et al., 2010). *S. pombe* cells were transformed using lithium acetate and heat shock (Moreno et al., 1991). Selection was performed on YES agar plates containing G418 (Sigma), nourseothricin (HKI Jena), hygromycin B (Roche) or minimal media (EMM) agar plates lacking uracil.

#### Generation of Vps24, Vps4 and Cmp7 Functional Tagged Constructs

The following constructs were designed with restriction sites flanking the 3’overlap region, endogenous promoter, ORF, tag and terminator: XhoI-3’UTR-SrfI-promoter-Vps4-AscI-3xHA-eGFP-PacI-terminator-SacII, XhoI-3’UTR-EcoRV-promoter-Vps24-AscI-LAP-eGFP-PacI-terminator-XbaI, EcoRV-3’UTR-SrfI-promoter-Cmp7-AscI-LAP-eGFP-PacI-terminator-XbaI. Constructs were synthesised by Eurofins. The eGFP of the Cmp7 construct was switched to mNeonGreen using Gibson assembly (NEB). Each construct was cloned into a pJK210-based vector for transformation into *S. japonicus*. Plasmids were linearized with SrfI or EcoRV respectively and transformed into yeast, replacing the endogenous allele. The *vps4*^*EQ*^ and *vps4*^*ts*^ alleles were constructed by site-directed mutagenesis PCR based on corresponding budding yeast mutations (Babst et al., 1997).

#### Canavanine Sensitivity Assay for Functionality of ESCRT-III/Vps4 Constructs

ESCRT mutants show inhibited growth in the presence of the arginine analogue canavanine (Lin et al., 2008). We titrated canavanine concentration in EMM plates with supplements from 0-10 μg ml^-1^ and determined the optimal working concentration for *S. japonicus* to be around the 4-4.5 μg ml^-1^ level. Note that this is higher than the working concentration for *S. cerevisiae*. *vps4-3xHA-GFP* and *vps24-LAP-GFP* strains grew similar to the WT when exposed to 4.5 μg ml^-1^ canavanine (Figure S2A). This suggests that the MVB pathway and therefore the ESCRT machinery is functional in these cells.

#### FM4-64 Staining

Cells were grown to early exponential growth phase. 10 ml of cells were spun down at 2103 xg for 1 minute and resuspended in 100 μl YES medium. 1 μl of FM4-64 (Thermo Fisher, stock 1 mg/ml in DMSO) was added and incubated with cells for 5 minutes at 30°C. Subsequently, the cells were washed twice with YES and resuspended in 10 ml of YES and grown for 1 hr at 30°C in a shaking incubator before imaging to allow uptake of the dye and transport to the vacuoles.

#### Vps4 Inactivation Using a Temperature-Sensitive Allele

Cells were grown in liquid culture overnight at 25°C in a shaking incubator to early exponential growth phase (OD_595_ = 0.2). The culture was shifted to 36°C and samples were taken at time 0 and then at 1, 2 and 4 hours in preheated tubes. Slides for imaging were prepared on a heat block at 36°C. Imaging was performed in a pre-heated chamber at 36°C.

#### Colony-Forming Unit (CFU) Assay

Cells were grown overnight at 30°C in a shaking incubator. The next day, cells with an OD_595_ of 0.2-0.3 were diluted to 0.1. Next, three serial dilutions of 10x, 100x and 1000x were prepared. Of these dilutions, triplicates of 50 μl were plated on YES plates and spread using glass beads. After two days of incubation at 30°C the plates were scanned using an Epson Perfection V700 Photo scanner and Epson Scan software. CFU count and size were analysed using ImageJ.

#### Microscopy

For imaging, cells grown in liquid YES media to early exponential growth phase were placed on a thin YES 2% agarose strip and immobilised by a cover slip, which was sealed with wax. This slide was first rested for 30 minutes at the 30°C. During imaging cells where kept at 30°C in an environmental control chamber. Imaging was performed on a Nikon Eclipse Ti-E inverted system equipped with CSU-X1 spinning disk confocal unit and 600 series SS 488nm, SS 561nm lasers. Images were obtained with an Andor iXon Ultra U3-888-BV monochrome EMCCD camera using a Nikon CFI Plan Apo Lambda 100x/1.45NA objective lens. Images presented in this report are Z-projections unless noted otherwise. For microscopy images contrast and brightness were adjusted for each individual image for optimal visibility unless noted otherwise. For time-lapse imaging, laser power and exposure time were adjusted to minimise photobleaching.

#### Western Blotting of Vps4-GFP

Cells were grown until exponential growth phase. 5 OD_595_ equivalents were pelleted for 1 min at 2103 xg and liquid medium was removed. Cells were resuspended in 1 ml ice-cold dH_2_O and transferred to 1.5 ml Eppendorf tubes. Cells were washed and resuspend in 1 ml cold dH2O. Next, 110 μl ice-cold TCA was added and proteins were precipitated for 1 hour on ice. Lysates were pelleted for 10 minutes at 18213 xg at 4°C in an Eppendorf centrifuge. Supernatant was removed and the pellet was resuspended in 1 ml ice-cold acetone. Lysates were washed once. After removing the supernatant, pellets were dried in a speed-vac (Eppendorf) for 2 minutes at room temperature. Pellets were resuspended in 300 μl boiling buffer (50 mM Tris pH 8.0, 1 mM EDTA, 1% SDS) and transferred to pre-chilled screw-cap tubes with zirconium beads. Lysates were disrupted using a MP Biomedicals cell disruptor for 2 x 15 sec at 4°C and 6.5 m/sec with 150 seconds of cooling down on ice in between. Lysates were extracted from the beads using a hot needle and span down for 3 min at 526 xg at 4°C. Debris were cleared by spinning down lysate at 1383 xg for 4 minutes at room temperature. Lysates were boiled at 95°C for 5 minutes. A 100 μl 4x 10% ß-mercaptoethanol sample buffer was added and lysates were heated at 65°C for 10 minutes. 20 μl of lysate (0.25 OD_600_ equivalent) was loaded per lane on a NU-PAGE 4-12% Bis-Tris gel (Invitrogen) and run at 180V for 1 hour. Proteins were transferred at 100V for 1 hour. For detecting Vps4-GFP PVDF membranes were blocked for 1 hour with blocking buffer and then incubated for 1 hour with mouse ⍺-GFP antibodies. Membranes were washed with 1% Tween-PBS and incubated for 1 hour in blocking buffer with IRDye800 conjugated anti-mouse antibodies. Total protein levels were detected using the LI-COR Revert 700 Total Protein stain kit. Proteins were detected using the Odyssey Infrared Imaging System (LI-COR Biosciences).

#### Western Blotting of Immunoprecipitated Lem2-GFP

Cells were grown until exponential growth phase. 30 OD_595_ equivalents were pelleted for 1 minute at 2103 xg and liquid medium was removed. Cells were washed once with dH_2_O and transferred to 1.5 ml Eppendorf tubes. Supernatant was removed and cell pellets were snap-frozen in liquid nitrogen. Pellets were either stored at -80°C or directly used. Cell pellets were thawed on ice, resuspended in 200 μl NP-40 lysis buffer (6 mM Na_2_HPO_4_, 4 mM NaH_2_PO_4_.H_2_O, 1% NONIDET P-40, 150 mM NaCl, 2 mM EDTA, 50 mM NaF, 4 μg/ml leupeptin, 0.1 mM Na_3_VO_4_) and transferred to pre-chilled screw-cap tubes with zirconium beads. Lysates were disrupted with a MP Biomedicals cell disruptor for 2 x 15 sec at 4°C and 6.5 m/sec with 150 seconds of cooling down on ice in between. Lysates were extracted from the beads using a hot needle and spinning down for 3 minutes at 526 xg at 4°C. Debris were cleared by spinning down lysate at 1383 xg for 4 minutes at 4°C. Lysates were incubated with GFP-trap beads (Chromotek) at 4°C for 4 hours or overnight. Beads were washed twice with lysis buffer. Protein was eluted using NuPage sample buffer (Invitrogen) at 95°C for 5 minutes. All protein was loaded on a NU-PAGE 4-12% Bis-Tris gel (Invitrogen) and run at 180V for 1 hour. Proteins were transferred at 100V for 1 hour. For detecting Lem2-GFP PVDF membranes were blocked for 1 hour with blocking buffer and then incubated for 1 hour with mouse ⍺-GFP antibodies. Membranes were washed with 1% Tween-PBS and incubated for 1 hour in blocking buffer with IRDye800 conjugated anti-mouse antibodies. Total protein levels were detected using the LI-COR kit. Detection was done using the Odyssey Infrared Imaging System (LI-COR Biosciences).

#### Protein Expression and Purification

Expression of proteins was performed as previously described (Babst et al., 1998, Teis et al., 2008). Recombinant proteins were expressed in C41(DE3) pLysS *E. coli* (Lucigen) and induced at 37°C for 4h in 1mM IPTG (Thermo R0392). GST-tagged proteins were purified with Glutathione Sepharose 4B (GE Healthcare, GE17-0756-01), washed and either eluted with glutathione or cleaved with PreScission protease (GE Healthcare, 27084301) overnight at 4°C. Recombinant proteins were dialyzed in a Slide-a-lyzer (VWR, 514-0172) overnight in ATPase buffer (100mM potassium acetate, 5mM magnesium acetate, 20 mM Hepes 7.4) and were additionally purified via a Superdex 2000 column (GE Healthcare). Proteins were flash frozen in liquid nitrogen and stored at -80°C until further usage. Plasmids used for protein expression are listen in the Key Resources Table.

#### GST Pulldown Assays

GST pulldown assays were performed as previously described (Shestakova et al., 2010, Adell et al., 2014). Pierce magnetic glutathione beads (Thermo, 78602) were incubated in 0.1% BSA in ATPase buffer overnight. 5μg of GST-tagged proteins were bound to beads for 2 hours at 4°C, washed and optionally incubated with 500 ng of Cmp7-3xFLAG and/or Vps32-3xMyc for 1 hour at 4°C, followed by 5 five washing steps in ATPase buffer (+/- ATP) with 600mM NaCl. 500 ng of Vps4^E233Q^ -3xHA or Vps4-3xHA was added in the presence or absence of 1mM ATP for 10 minutes at room temperature. After three washing steps in ATPase buffer with 300mM NaCl, proteins were eluted from beads using sample buffer (2% SDS, 100mM Tris 6.8, 10% glycerol, 5% beta-mercaptoethanol, 0.01% bromophenol blue) at 96°C for 10 minutes. Samples were separated on a 12.5% SDS Page. Proteins were either stained by Brilliant Blue Coomassie or subjected to Western blotting.

#### Chromatin-Immunoprecipitation and qPCR

Chromatin-immunoprecipitation (ChIP) was performed broadly according to the protocol of (Barrales et al., 2016). The following buffers were used. ChIP lysis buffer: 50 mM HEPES/KOH pH 7.5, 140 mM NaCl (500 mM in high salt lysis buffer), 1 mM EDTA, 1% Triton X-100, 0.1% Na-deoxycholate. Wash buffer: 10 mM Tris-HCl pH 8.0, 250 mM LiCl, 1 mM EDTA, 0.5% NP-40, 0.5% Na-deoxycholate. Elution buffer: 50 mM, Tris/HCl, pH 8.0, 10 mM EDTA, 0.8% Na-deoxycholate. TE: 10 mM Tris/HCl pH 7.5 ,1 mM EDTA.

For cross-linking, cells were grown in YES medium overnight at 30°C until an OD_595_ of 0.3-0.4 was reached. 60 ODs and 15 ODs of cells were used for Lem2-GFP and H3K9me2 ChIP, respectively. Cells were crosslinked with fresh 37% formaldehyde (FA) for 10 minutes at room temperature: 2.7 ml of FA per 100 ml cells for Lem2-GFP ChIP and 1.35 ml of FA per 50 ml cells for H3K9me2 ChIP. FA was quenched by adding 2.5M glycine: 5 ml for Lem2-GFP ChIP and 2.5 ml for H3K9me2 ChIP. Cells were spun down at 2103 xg for 1 min and washed twice with 25 ml of ice-cold phosphate-buffered saline (PBS). Cells were resuspended in 800 μl ice cold PBS and transferred to a 1.5 ml Eppendorf tube and spun down at 845 xg for 1 minute. Supernatants were removed and cells were snap-frozen in liquid nitrogen. From here, cell pellets were either stored at -80°C or used for IP.

Cell pellets were then thawed on ice. From here on until reverse crosslinking all steps were at 4°C. Note that for Lem2-GFP two pellets of 30 ODs were used throughout the protocol and combined during DNA purification as the Lem2 extraction protocol was optimised for this amount of cells. 10 ml of ChIP lysis buffer was chilled and prepared with final concentrations of 1 mM AEBSF, 100 μg/ml leupeptin and Roche complete protease inhibitor cocktail. 500 ml of lysis buffer with protease inhibitors was added to each cell pellet. Pellets were dissolved and added to screw-cap tubes with zirconium beads. Cells were disrupted in a MP Biomedicals cell disruptor for 2 cycles of 15 seconds with 150 seconds on ice in between. Using a hot needle, a hole was poked in each tube and tubes were spun down for 3 min at 526 xg, while resting on a pipette tip to collect the lysates. The lysates were sonicated in a Digenotide Bioruptor Plus (55 cycles of 30 seconds on/off for Lem2-GFP and 45 cycles of 30 seconds on/off for H3K9me2). Cells were spun down at 16000 xg for 10 min. The supernatant was transferred to a new pre-chilled tube. Volumes were adjusted to 540 μl with lysis buffer containing protease inhibitors.

For Lem2-GFP ChIP 5 μg of ⍺-GFP antibodies was added to each tube. For H3K9me2 ChIP 2 μl of ⍺-H3K9me2 antibody was added to each sample. Samples were incubated overnight on a rotator at 10 rpm. Following day, 60 μl of each sample were taken to be used as input (Lem2-GFP samples were combined first and divided in two again after). To each input sample 140 μl of TE-1% SDS was added. Next, 15 μl of protein-G dynabeads were added and incubated for another 4 hours.

Beads were washed twice with 500-μl lysis buffer, twice with 500 μl high salt lysis buffer, twice with 500 μl wash buffer and transferred to a new tube in 150 μl TE. Beads were washed one final time and resuspended in 200 μl elution buffer. Washed IP samples and input samples were eluted for 10 min at 95°C in a shaking heat block at 1400 rpm. Samples were spun down to remove condensation from the lid. Reversal of crosslinking was performed for 3 hours at 65°C. Samples were incubated for 1 hour with 4 mg/ml Proteinase K at 55°C, spun down to remove condensation from the lid, resuspended and incubated for another hour. Samples were spun down to collect the beads and the supernatant was transferred to a new tube. DNA purification was performed using the Zymo Research, ChIP DNA Clean & Concentrator™ kit. DNA was collected in 17 μl of water.

For qPCR, each IP and input sample were diluted 100x. qPCR on Lem2-GFP samples was performed using SyGreen Blue Mix Hi-ROX master mix and qPRC on H3K9me2 were performed using Powerup SYBR Green master mix according to manufacturer’s protocol. qPCR was performed in 384 well plates with two technical repeats per primer pair per strain. For each experiment centromeric loci and telomeric loci were tested in individual plates. Plates were run on an Applied Bioscience Quantstudio 6 Flex real-time PCR system and data was extracted and analysed with associated software. Primers for qPCR are listed in Table S2.

### Quantification and Statistical Analysis

#### Analysis Software

All imaging data was analysed and imaging data figures were prepared in ImageJ (Schindelin et al., 2012, Schindelin et al., 2017). Data was analysed using Microsoft Excel or R (R core team, 2019). Statistics was performed in Graphpad Prism. Graphs were prepared in Graphpad Prism. Figures were prepared in Adobe Indesign or Adobe Illustrator.

#### NE Resealing Assay

For imaging of NE resealing cells undergoing mitosis were typically imaged every 30 seconds for a maximum duration of 40 min (n = 15 cells). Z-stacks of slices with 0.5 μm distance (total stack 6.0 μm) were obtained at each time point. Maximum Z-projections were made and the average intensity of a circle with a diameter of 0.695 μm (10 pixels) was measured in the nucleus next to the SPB (used as a spatial cue).

#### Analysis of Recruitment of Vps4 to Distal NE Tails

For time-lapse imaging, cells were imaged every 1 minute for a maximum duration of 30 minutes. Z-stacks of slices with 0.5 μm distance (total stack 4.5 μm) were obtained at each time point. Maximum Z-projections were made and a line was drawn of 8 pixels wide and 80 pixels long starting from the SPB to the end of the NE ‘tail’ structure. Fluorescence intensity was extracted using the plot-profile function for 10 cells (Figure 2C). For Figure 2D (timing of Vps4 recruitment to the ‘tails’) 13 cells were followed from NE rupture to spindle breakdown.

#### Recruitment of ESCRT-III/Vps4 to the NE during

For imaging of persistent recruitment of Vps4-GFP to the NE (Figures S4B and S4C), cells were imaged every 10 seconds for 5 minutes. Z-stacks of slices with 0.5 μm distance (total stack 4.5 μm) were obtained at each time point. One replicate experiment constituted imaging several fields of view for a total period of 1 hour and was repeated three times. For analysis, single confocal slices of each cell at each time-point were screened for fluorescence signal at the NE. The ImageJ line-plot tool was used to confirm if the fluorescence signal was an ESCRT-III/Vps4 event directly on the NE marked by Nup189-mCherry. Persistent events were defined as events that lasted from the start to the end of each 5-minute time-lapse.

#### Quantification of Dispersed and Clustered Lem2 in ESCRT-III/Vps4 Deficient Cells

For the quantification of Lem2-mCherry signal in the experiments of Figures 4D and 4F Z-stacks of slices with 0.5 μm distance (total stack 4.5 μm) were obtained. Z-slices through the middle of the cells were used for quantification and the Nup189-GFP and Sad1-mNeonGreen signals were used as guides for the location of the NE in the absence of dispersed Lem2-mCherry signal. In ImageJ, the mean intensity of a circular selection of 8 by 8 pixels was used to measure the intensity of individual clusters and the dispersed signal. For each cell a background measurement was taken. For each time-point background measurements were averaged and used to normalise Lem2-mCherry signal.

#### ChIP-qPCR Analysis

Data was exported from the Applied Bioscience Quantstudio 6-7 Flex Studio software to Excel. Technical repeats were averaged. IP was corrected as percentage of input for each biological repeat (n = 3). For the input samples the adjusted cycle-threshold (CT) value was calculated using the following function: adjustedCT = CT - log_2_(dilution factor). Next, the % of input was calculated with the following function: % of input = 100^∗^2ˆ(deltaCT), where deltaCT is the CT value of the test sample minus the adjusted CT value.

R was used to generate the data of Figures 5F–5I. Figure 5I was generated by comparing the mean of the biological repeats for each genomic locus of the Lem2-GFP ChIP in the *vps4*Δ background to the H3K9me2 ChIP in the WT background. Correlation analysis was performed with the Pearson's correlation test and a graph was generated with each data point, the linear trendline and the 95% confidence interval. For Figures 5F–5H only the genomic loci with higher than median levels of H3K9me2 signal were analysed. For each of these loci the mean Lem2-GFP signals in each of the genetic backgrounds were pooled. Similar analysis was performed specifically for centromeric and telomeric loci. A Kruskal-Wallis test followed by a Dunn’s post-hoc test and Benjamini-Hochberg false discovery rate correction was performed on each of these pooled data sets.

#### Measurement of Distance between Taz1 or Mis6 and the NE

Z-stacks of slices with 0.25 μm distance (total stack 4.5 μm) were obtained. To measure the distance of a Taz1 or Mis6 focus to the NE a line was drawn in a single Z-slice from the centroid of Taz1/Mis6 signal to the centroid of the NE signal and the length in pixels was measured and amplified by 0.0695 to obtain the physical distance. In many cases the focus was nearer to the NE in Z than in Y or X. In this case the orthogonal view function of ImageJ was used to count the number of Z-steps from the centre of the focus to the centre of the NE signal as the distance (resulting in a lot of data points having 0.25, 0.5, 0.75 etc. as distance).

### Data and Code Availability

The data that support the findings of this study are available from the corresponding authors upon request. The authors declare that all data reported in this study are available within the paper and its Supplemental Information files.

## Supplemental Information

Document S1. Figures S1–S6

Table S1. List of *S.* *japonicus* and *S.* *pombe* Strains Used in This Study, Related to STAR Methods

Table S2. List of Primers Used in This Study, Related to STAR Methods TF, Thermo Fisher Scientific. IDT, Integrated DNA Technologies, Inc (Coralville, IA, USA).

Document S2. Article plus Supplemental Information
