# Supplementary material for: ESCRT-III/Vps4 Controls Heterochromatin-Nuclear Envelope Attachments
Source: Dev Cell. 2020 Apr 6;53(1):27–41.e6. doi: 10.1016/j.devcel.2020.01.028 (PMC7139201; doi:10.1016/j.devcel.2020.01.028)
Supplement: Document S1. Figures S1–S6 [file mmc1.pdf]

Developmental Cell, Volume *53*

Supplemental Information

ESCRT-III/Vps4 Controls Heterochromatin-Nuclear Envelope Attachments

Gerard H. Pieper, Simon Sprenger, David Teis, and Snezhana Oliferenko

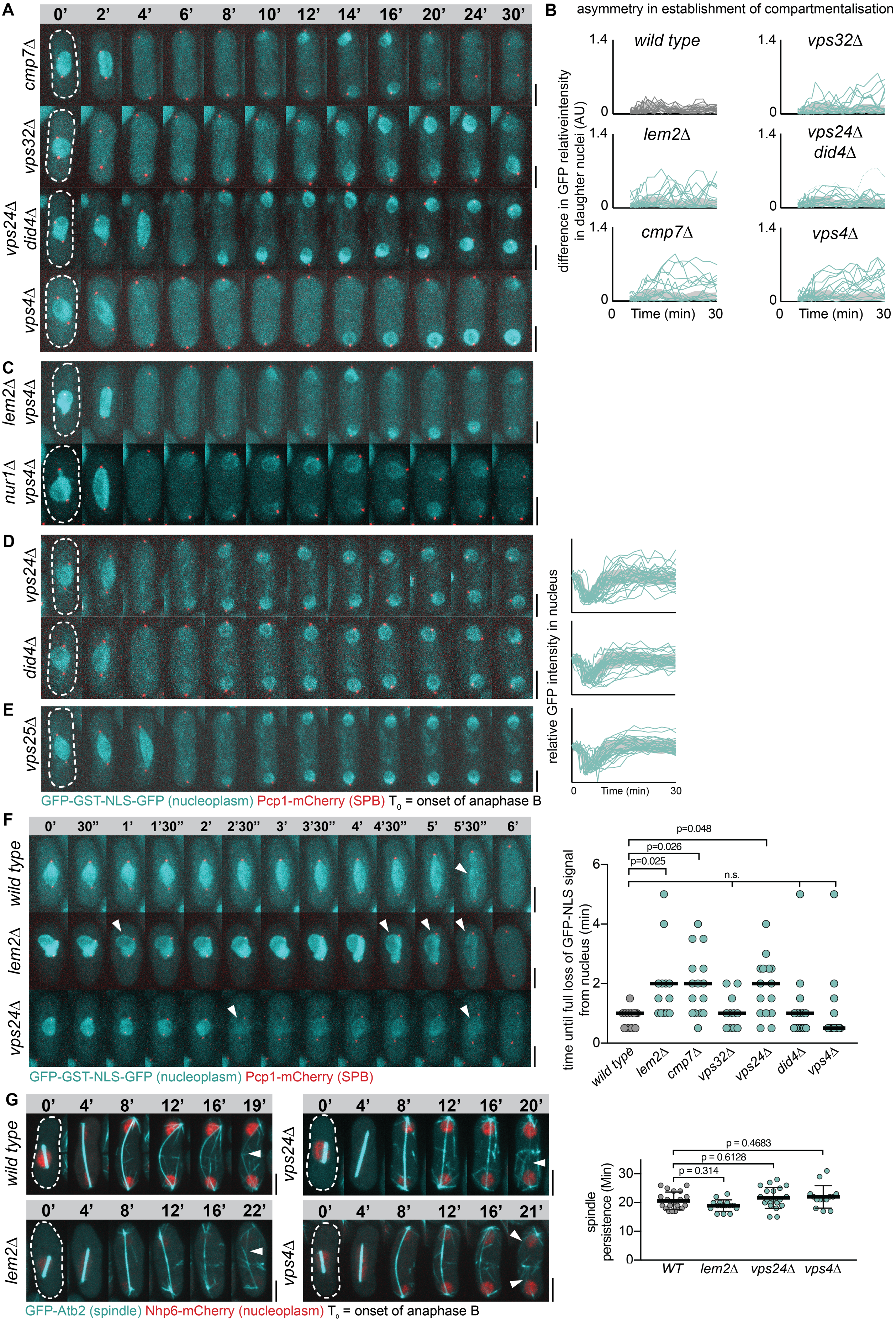

A
0' 2' 4' 6' 8' 10' 12' 14' 16' 20' 24' 30'
cmp7Δ
vps32Δ
vps24Δ did4Δ
vps4Δ
B
asymmetry in establishment of compartmentalisation
difference in GFP relativeintensity in daughter nuclei (AU)
wild type
vps32Δ
lem2Δ
vps24Δ did4Δ
cmp7Δ
vps4Δ
1.4
0
Time (min)
30
C
lem2Δ vps4Δ
nur1Δ vps4Δ
D
vps24Δ
did4Δ
E
vps25Δ
relative GFP intensity in nucleus
Time (min)
GFP-GST-NLS-GFP (nucleoplasm) Pcp1-mCherry (SPB) T_0 = onset of anaphase B
F
0' 30" 1' 1'30" 2' 2'30" 3' 3'30" 4' 4'30" 5' 5'30" 6'
wild type
lem2Δ
vps24Δ
GFP-GST-NLS-GFP (nucleoplasm) Pcp1-mCherry (SPB)
time until full loss of GFP-NLS signal from nucleus (min)
p=0.048
p=0.026
p=0.025
n.s.
wild type
lem2Δ
cmp7Δ
vps32Δ
vps24Δ
did4Δ
vps4Δ
G
0' 4' 8' 12' 16' 19'
wild type
0' 4' 8' 12' 16' 20'
vps24Δ
0' 4' 8' 12' 16' 22'
lem2Δ
0' 4' 8' 12' 16' 21'
vps4Δ
GFP-Atb2 (spindle) Nhp6-mCherry (nucleoplasm) T_0 = onset of anaphase B
spindle persistence (Min)
p = 0.4683
p = 0.6128
p = 0.314
WT
lem2Δ
vps24Δ
vps4Δ

Figure S1. Nucleocytoplasmic compartmentalization during mitosis is affected in the absence of ESCRT-III/Vps4 function. Related to Figure 1. (**A**) Representative GFP-NLS Pcp1-mCherry-expressing cells of indicated genotypes used for the quantification in Fig. 1G. (**B**) Quantification of the asymmetry in the re-establishment of compartmentalisation. Shown is the absolute difference in GFP-NLS fluorescence intensity between the two daughter nuclei from the 5-minute timepoint. The same cells as in Fig. 1F-G were used for quantification. (**C**) Representative GFP-NLS Pcp1-mCherry-expressing cells of indicated genotypes used for the quantification in Fig. 1H. (**D**) Time-lapse images of representative GFP-NLS Pcp1-mCherry-expressing cells of indicated genotypes. Shown is the quantification of GFP-NLS fluorescence signal as in Fig. 1F. (**E**) Same as (**D**) but for the *vps25Δ* (ESCRT-II) mutant. (**F**) Premature loss of nuclear integrity during mitosis in *lem2Δ* and ESCRT-III/Vps4 mutants. Arrowheads indicate the onset of full loss of GFP-NLS signal from the nucleus. Note the single abrupt rupture in the WT versus the repeated ruptures and slow leaking of the nucleoplasm in the mutants. The same set-up as in Fig. 1 was used to quantify time until full loss of GFP-NLS signal from the nucleus after anaphase onset. Medians are indicated. p-values determined by Kruskal-Wallis multiple comparison test. (**G**) Time-lapse maximum projection sequences of GFP-Atb2 and Nhp6-mCherry-expressing cells of indicated genotypes starting from the onset of anaphase B. Arrowheads indicate the breakage of the spindle. Quantification of time between anaphase B onset and breakage of the spindle is presented on the *right* ($n \geq 14$ cells; p-values determined by one way ANOVA followed by Dunnett's comparison test. (**A**, **C**-**G**) Scale bars represent 5 μm.

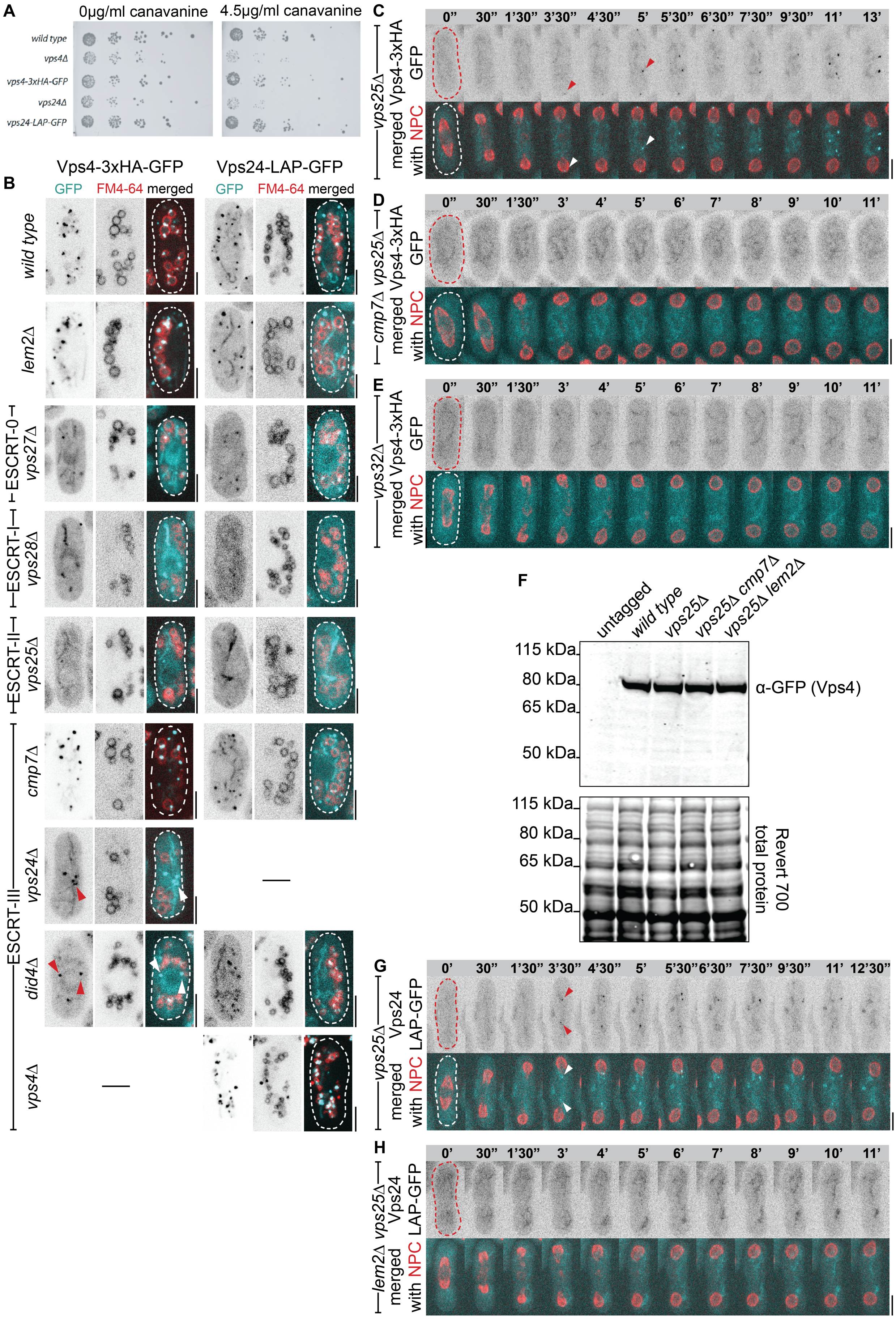

A
0µg/ml canavanine
4.5µg/ml canavanine
wild type
vps4Δ
vps4-3xHA-GFP
vps24Δ
vps24-LAP-GFP
B
Vps4-3xHA-GFP
Vps24-LAP-GFP
GFP FM4-64 merged
wild type
lem2Δ
ESCRT-0
vps27Δ
ESCRT-I
vps28Δ
ESCRT-II
vps25Δ
ESCRT-III
cmp7Δ
vps24Δ
did4Δ
vps4Δ
C
vps25Δ
Vps4-3xHA GFP
merged with NPC
0" 30" 1'30" 3'30" 4'30" 5' 5'30" 6'30" 7'30" 9'30" 11' 13'
D
cmp7Δ vps25Δ
Vps4-3xHA GFP
merged with NPC
0" 30" 1'30" 3' 4' 5' 6' 7' 8' 9' 10' 11'
E
vps32Δ
Vps4-3xHA GFP
merged with NPC
0" 30" 1'30" 3' 4' 5' 6' 7' 8' 9' 10' 11'
F
untagged
wild type
vps25Δ
vps25Δ cmp7Δ
vps25Δ lem2Δ
115 kDa
80 kDa
65 kDa
50 kDa
α-GFP (Vps4)
Revert 700 total protein
G
vps25Δ
Vps24 LAP-GFP
merged with NPC
0' 30" 1'30" 3'30" 4'30" 5' 5'30" 6'30" 7'30" 9'30" 11' 12'30"
H
lem2Δ vps25Δ
Vps24 LAP-GFP
merged with NPC
0' 30" 1'30" 3' 4' 5' 6' 7' 8' 9' 10' 11'

Figure S2. Subcellular localization of Vps4 and Vps24. Related to Figure 2. (**A**) Canavanine sensitivity assay. WT, *vps4Δ*, and *vps24Δ* mutant cells or cells expressing Vps4-GFP or Vps24-GFP were spotted on EMM plates containing either 0 or 4.5 µg ml^{-1} canavanine. Note that ESCRT-III tagging does not render cells sensitive to canavanine. (**B**) Shown are single confocal slices of cells expressing Vps4-GFP or Vps24-GFP that were incubated with FM4-64 for 5 minutes followed by washout of the drug and recovery for 1 hour before imaging. Note that in early ESCRT mutants Vps4 and Vps24 are no longer recruited to FM4-64 marked structures. Arrowheads indicate bright Vps4 foci at the NE in *vps24Δ* and *did4Δ* mutants. (**C**) A time-lapse sequence of a *vps25Δ* Vps4-GFP Nup189-mCherry-expressing cell starting prior to NE rupture (n = 12). Note that the NPCs are excluded from the 'tails'. (**D**) A time-lapse sequence of a *cmp7Δ vps25Δ* Vps4-GFP Nup189-mCherry cell starting prior to NE rupture (n = 15). (**E**) A time-lapse sequence of a *vps32Δ* Vps4-GFP Nup189-mCherry cell starting prior to NE rupture (n =12). (**F**) Western blot of Vps4-GFP in strains of indicated genotypes used for imaging (n = 3 experiments). Revert 700 stain was used to visualize total protein loading. (**G**) A time-lapse sequence of a *vps25Δ* Vps24-GFP Nup189-mCherry-expressing cell starting prior to NE rupture (n = 12). Note that Vps24 also localizes to the 'tails' despite not being required for re-establishment of nucleocytoplasmic compartmentalisation. (**H**) A time-lapse sequence of a *lem2Δ vps25Δ* Vps24-GFP Nup189-mCherry-expressing cell starting prior to NE rupture (n = 8) shows the dependency of Vps24 on Lem2 for NE recruitment. (**B**-**E**, **G**-**H**) Shown are Z-projections of spinning disk confocal stacks, unless indicated otherwise. Scale bars represent 5 µm.

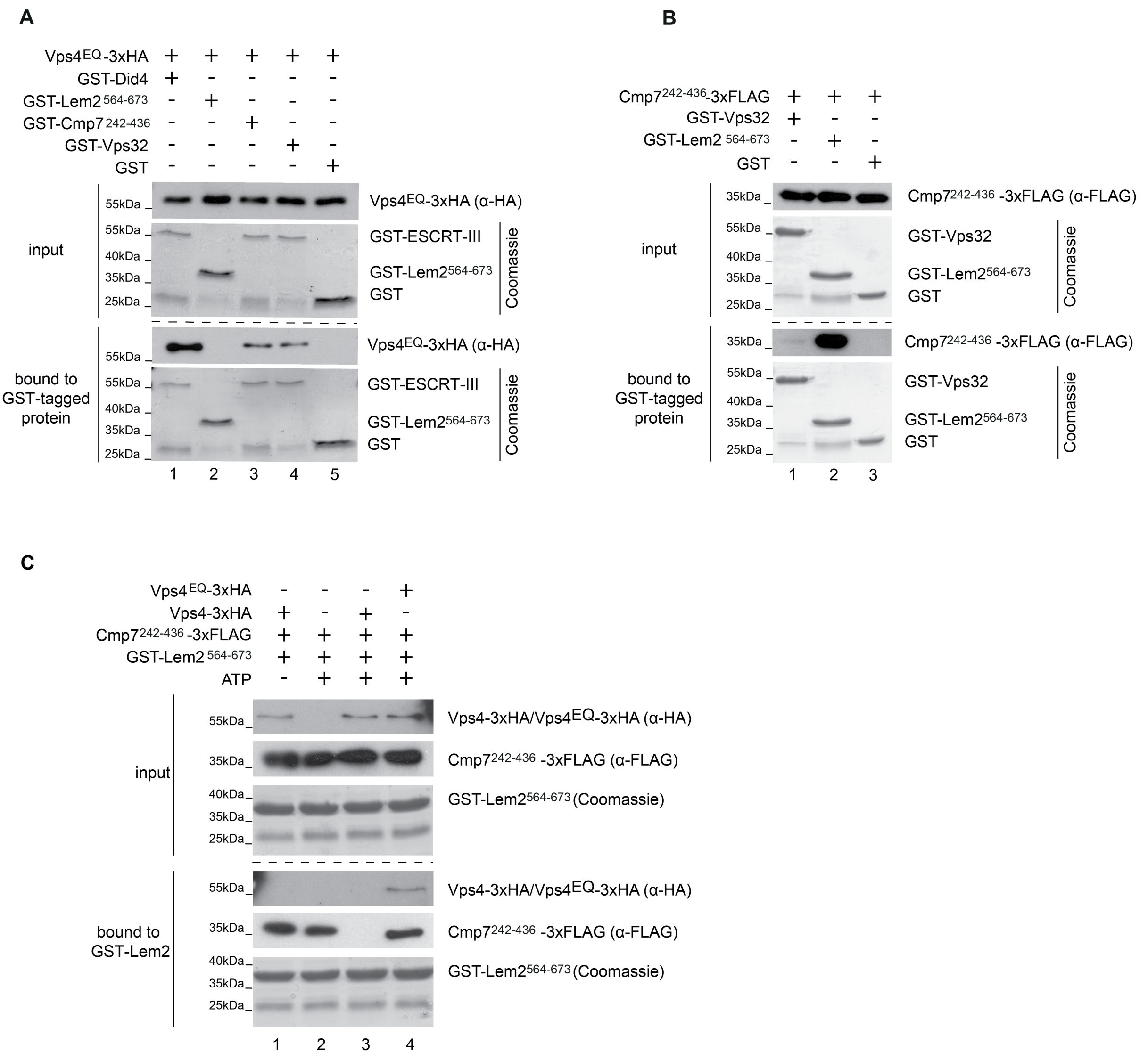

Figure S3. In vitro interactions between Lem2 and ESCRT-III/Vps4. Related to Figure 3. (**A**) *In vitro* binding experiment. An ATPase deficient Vps4^{EQ} that can bind to its substrates but cannot hydrolyse ATP was added to glutathione beads coated with GST-Did4, GST-Lem2$^{564-673}$, GST- Cmp7$^{242-436}$ or GST-Vps32. Vps4^{EQ} bound strongly to Did4 as expected and weakly to Cmp7$^{242-436}$ and Vps32. No binding of Vps4^{EQ} to Lem2$^{564-673}$ could be detected. (**B**) *In vitro* binding experiment where Cmp7$^{242-436}$ was added to glutathione beads coated with either GST-Vps32, GST-Lem2$^{564-673}$ or GST. Note that Cmp7$^{242-436}$ binds to Lem2$^{564-673}$ but not efficiently to Vps32 alone. (**C)** An *in vitro* binding experiment using a setup similar to Fig. 3A. Lem2$^{564-673}$- Cmp7$^{242-436}$ complexes were incubated with either WT Vps4 or Vps4^{EQ}, in the presence or absence of ATP. WT Vps4 could only disassemble the Lem2-Cmp7 complexes in the presence of ATP (lane 3), but not when ATP was absent (lane 1).

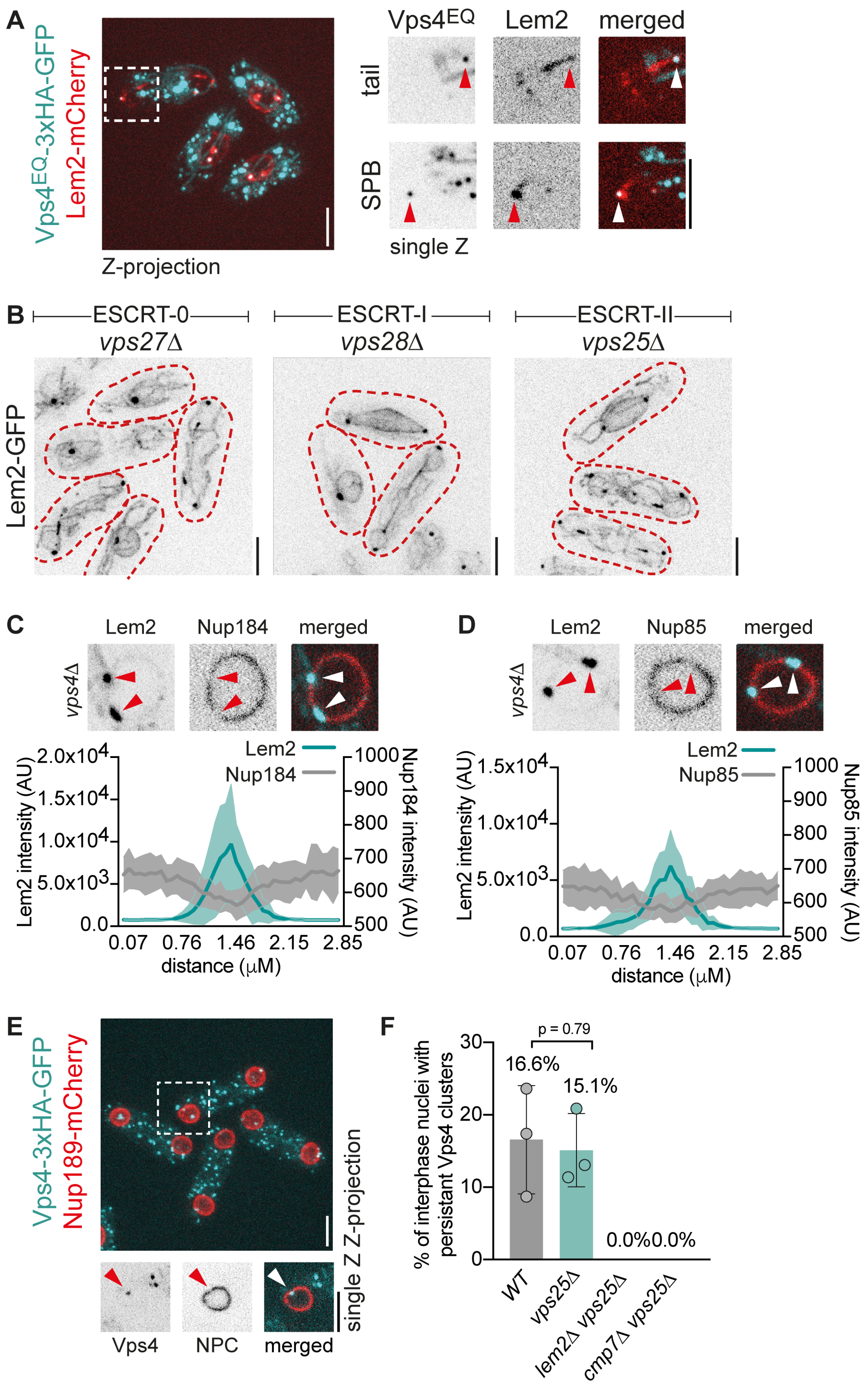

Figure S4. Lem2 clusters in Vps4-deficient *S. japonicus* do not represent SINCs described in budding yeast. Related to Figure 4. (A) Representative image of cells co-expressing Vps4^{EQ}-GFP and Lem2-mCherry. Magnified images on the right focus on enrichment of Vps4^{EQ}-GFP at the distal end of Lem2-mCherry 'tail' and the SPB. Arrowheads indicate colocalisation of Vps4^{EQ}-GFP and Lem2-

mCherry. Note that Lem2 is only partially clustered in this mutant. (**B**) Maximum projection images of spinning disk confocal stacks of cells of indicated genotypes expressing Lem2-GFP. Note that Lem2 does not cluster at the NE in early ESCRT mutants. (**C**) Single confocal slices of nuclei of representative Nup184-mCherry Lem2-GFP *vps4*Δ cells. Arrowheads indicate dips in NPC fluorescence signal intensity corresponding to Lem2-GFP clusters. Shown is the quantification of the Lem2-GFP and Nup184-mCherry signals centred on Lem2 clusters (n = 20, solid line represents mean and shaded colours represent standard deviation). (**D**) Same setup as in (**C**) but for Nup85-mCherry. (**E**) Representative Z-projection of a spinning disk confocal image of cells expressing Vps4-GFP and Nup189-mCherry. A magnified image below shows a nucleus with a persistent Vps4-GFP focus at the NE. (**F**) A graph showing the percentage of interphase cells of indicated genotypes harbouring a persistent Vps4-GFP focus at the NE (n = 3 experiments with at least 40 cells counted for each experiment; error bars indicate standard deviation; p-values determined by Student's t-test). Note that no persistent clusters were observed in *lem2*Δ or *cmp7*Δ mutants. (**A**-**E**) Scale bars represent 5 μm.

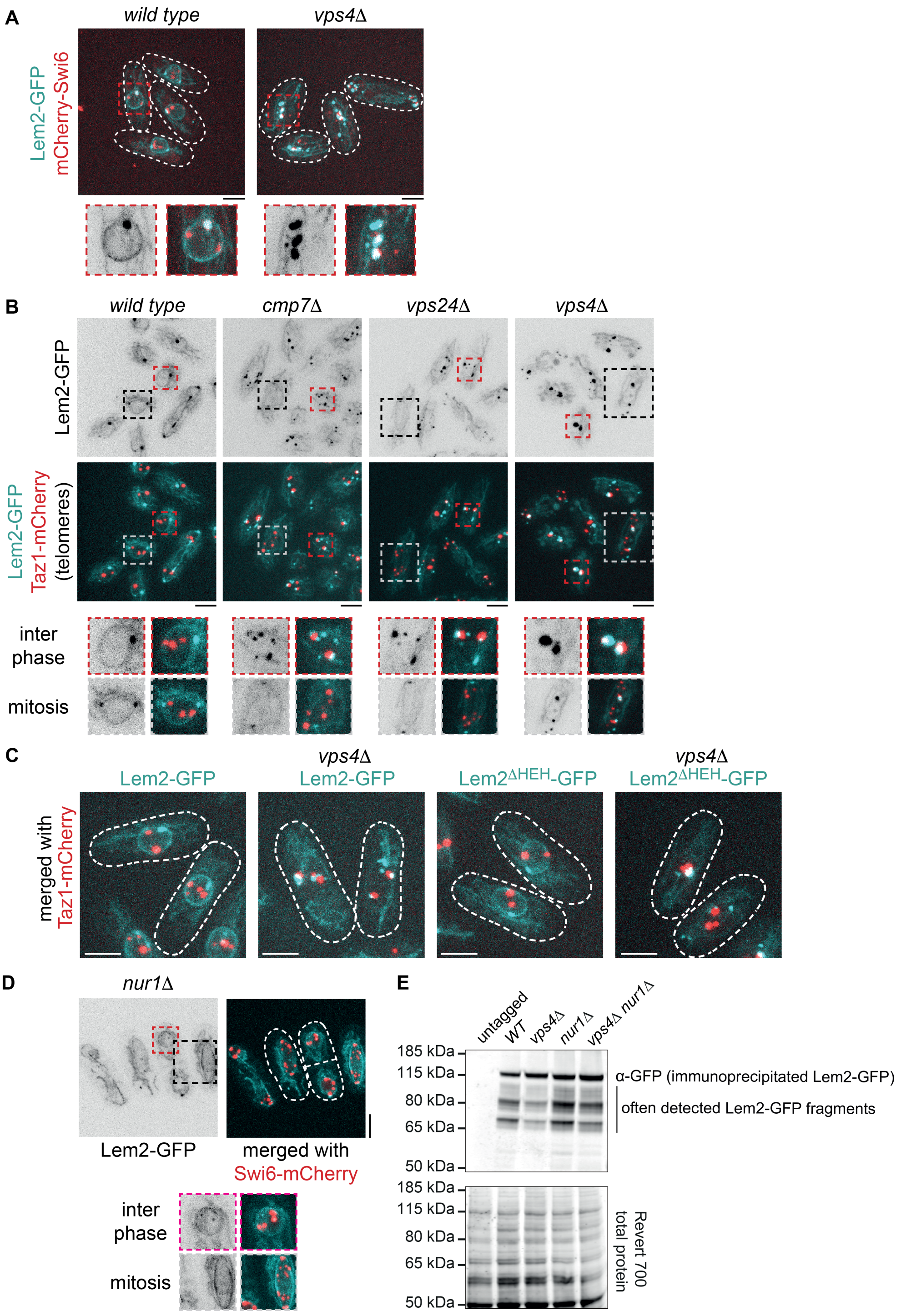

Figure S5. Lem2 clusters at heterochromatin in the absence of ESCRT-III/Vps4 function, independent of its HEH/LEM domain. Related to Figure 5. (**A**) A maximum projection of a spinning disk confocal stack of Lem2-GFP mCherry-Swi6-expressing cells of indicated genotypes. Magnifications of indicated areas are shown below. The N-terminally tagged mCherry-Swi6 is expressed from its native promoter.

(**B**) Spinning disk confocal Z-stacks of cells expressing Lem2-GFP and Taz1-mCherry in the indicated genetic backgrounds. Presented below are magnifications of interphase and mitotic cells. (**C**) Spinning disk confocal Z-stacks of cells expressing Lem2-GFP or Lem2$^{\Delta HEH}$-GFP either in the WT or the *vps4Δ* genetic background. Two colour overlays are shown. (**D**) A maximum projection of a spinning disk confocal stack of *nur1Δ* cells expressing Lem2-GFP and Swi6-mCherry, with the layout as in Fig. 5A. (**E**) Western blot of immunoprecipitated Lem2-GFP in the same strains used for the ChIP-qPCR experiments, shown in Fig. 5E-I. (**A**-**D**) Scale bars represent 5 μm.

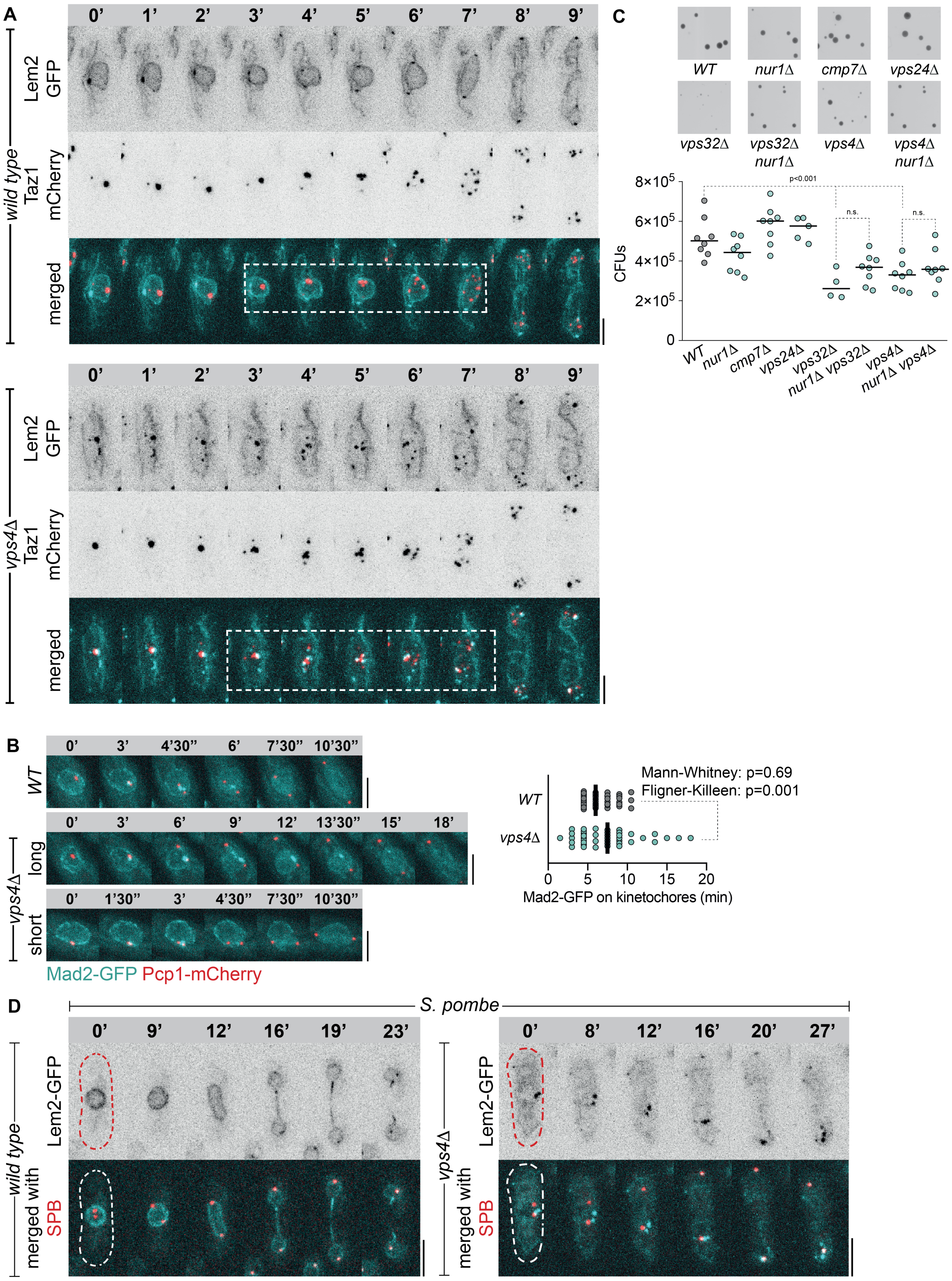

Figure S6. Chromosome arms exhibit persistent association with the NE in mitotic Vps4-deficient fission yeast cells. Related to Figure 6. (A) Full time courses and images of entire cells presented in Fig. 6C. (**B**) *Left*, time-lapse maximum projection sequences of representative cells of indicated genotypes

expressing Mad2-GFP and Pcp1-mCherry. *Right*, quantification of the duration of Mad2-GFP presence at kinetochores in the WT (n=40 cells) and *vps4Δ* (n=41 cells) genetic background. p-values determined by the Mann-Whitney U test and Fligner-Killeen test for homogeneity of variances. (**C**) CFU assay. (*Top*) representative images of colonies of each mutant. (*Bottom*) quantification of CFUs for the indicated genotypes for the same experiment as in Fig. 6D. p-values determined by the one-way ANOVA, Tukey's multiple comparisons test. (**D**) Maximum projections of time-lapse spinning disk confocal stacks of *S. pombe* cells of indicated genotypes expressing Lem2-GFP and Pcp1-mCherry. Note that Lem2 clusters do not disassemble in mitotic *vps4Δ* cells (n ≥ 7 cells). (**A**, **B**, **D**) Scale bars represent 5 μm.
